# Abscopal Effects in Metastatic Cancer: Is a Predictive Approach Possible to Improve Individual Outcomes?

**DOI:** 10.3390/jcm10215124

**Published:** 2021-10-31

**Authors:** Barbara Link, Adriana Torres Crigna, Michael Hölzel, Frank A. Giordano, Olga Golubnitschaja

**Affiliations:** 1 Department of Radiation Oncology, University Hospital Bonn, Rheinische Friedrich-Wilhelms-Universität Bonn, 53127 Bonn, Germany; Barbara.Link@ukbonn.de (B.L.); Adriana.Torres_Crigna@ukbonn.de (A.T.C.); Frank.Giordano@ukbonn.de (F.A.G.); 2 Institute of Experimental Oncology, University Hospital Bonn, Rheinische Friedrich-Wilhelms-Universität Bonn, 53127 Bonn, Germany; michael.hoelzel@ukbonn.de; 3 Predictive, Preventive, Personalised (3P) Medicine, Department of Radiation Oncology, University Hospital Bonn, Rheinische Friedrich-Wilhelms-Universität Bonn, 53127 Bonn, Germany

**Keywords:** predictive preventive personalized medicine (3PM), molecular patterns, abscopal effects, cancers, metastatic disease, radiotherapy, biomarker panel, immunotherapy, immune checkpoint inhibitors, multi-level diagnostics, liquid biopsy, prognosis, artificial intelligence, big data analysis, personalized treatment algorithms, individual outcomes, cost-efficacy

## Abstract

Patients with metastatic cancers often require radiotherapy (RT) as a palliative therapy for cancer pain. RT can, however, also induce systemic antitumor effects outside of the irradiated field (abscopal effects) in various cancer entities. The occurrence of the abscopal effect is associated with a specific immunological activation in response to RT-induced cell death, which is mainly seen under concomitant immune checkpoint blockade. Even if the number of reported apscopal effects has increased since the introduction of immune checkpoint inhibition, its occurrence is still considered rare and unpredictable. The cases reported so far may nevertheless allow for identifying first biomarkers and clinical patterns. We here review biomarkers that may be helpful to predict the occurrence of abscopal effects and hence to optimize therapy for patients with metastatic cancers.

## 1. Introduction

Radiotherapy (RT) has been widely used as an extremely effective anticancer treatment resulting in local tumor management. The targeted ionizing irradiation therapeutic effect is principally described to exert direct localized cell death through deoxyribonucleic acid (DNA) damage to promptly proliferating cells. However, preclinical studies have recognized irradiation to induce tumor recession at non-irradiated distant tumor milieus by means of tumor-specific immunity induction or immunogenic cell death (ICD) [1]. This systemic response is called the “abscopal effect” (AbE), derived from the Latin words *ab* (position away from) and *scopus* (mark or target), first introduced by Mole in 1953 and later enhanced by Andrews describing of distant normal tissue effects [2,3]. In modern oncology, AbE is becoming increasingly important as the association between and understanding of localized irradiation and immune-mediated systemic antitumor effects are enhanced. Nevertheless, the occurrence of AbE is mainly described in isolated case reports.

Here, we overview the present state of clinical data concerning AbE. The aim of this review is to provide an overview of the AbE in different cancer entities, along with its employment in combination with chemo- and immunotherapy and identify potential predictors/biomarkers associated with RT immunogenic features for patient stratification and treatment optimization.

## 2. Historical Background

The abscopal effect was first described and named by Mole in 1953 in a preclinical model of metastatic cancer [3]. Later his definition was broadened by Andrews to include radiation-related effects of both distant tumor and normal tissue [2,3]. Furthermore, Formenti et al. determined the abscopal response as a size shrinkage of 30% of a non-irradiated metastasis irrespective of other lesions [1]. AbE must be distinguished from bystander effects, the former being an effect that occurs at distance from the original radiation field, whereas the latter occurs as a local reaction due to radiation-induced signals from nearby irradiated cells [4].

Since its first description, AbE has been rarely observed in the clinical setting despite the millions of patients treated worldwide with local irradiation of tumor tissue in the absence of any systemic therapy. From 1969 to 2018, only 47 cases for multiple metastatic cancers including melanoma, renal cell carcinoma, lymphoma, breast cancer, and hepatocellular carcinoma following RT have been reported [5,6]. In Table 1, we provide case reports of AbE after RT in which no immunotherapy was utilized. We performed a literature search in PubMed up to March 2021, using as search criteria “abscopal effect” in the title and/or abstract and filtering for case reports. Results revealed nine additional AbE reports correlated with RT only.

The reported AbEs occurred independently from the patient age, treatment dose, fractionation, modality and characteristic of the target lesion. The cases varied widely in ethnicity and age (19–89 years), as well as in the applied radiation total doses (0.45–70 Gy) and doses per fraction (fr) (0.15–26 Gy). Most patients were treated with RT delivered in conventional fractionation (≤3 Gy), whilst only 14 cases received higher fraction doses. Approximately half of the reported cases were treated with RT for their primary site. AbE was observed ranging from two weeks up to several months (1–24 months) after receiving RT [5].

Postow et al. reported for the first time a case of AbE in a female patient that received systemic immunotherapy five years post primary tumor and lung metastasis treatment. Ipilimumab as immunotherapy against cytotoxic T-lymphocyte-associated protein 4 (CTLA-4) was administered to treat metastatic thoracic lesions, along with additional palliative RT. Thereby, five months following irradiation, non-targeted hilar and splenic metastatic lesions had regressed leading to minimal stable disease, and suggesting a correlation between AbE and the immune checkpoint blockade (ICB) [49]. With the introduction of the immune checkpoint inhibitor ipilimumab in clinical practice, the number of AbE related publications considerably increased. Adjepong et al. along with Dagoglu et al. thoroughly reviewed a total of 47 cases of AbE in patients with melanoma, lymphoma, renal cell carcinoma, pancreatic cancer, breast cancer, and prostate cancer, among others, treated with radiation in addition to immunotherapy in just six years between 2012 and 2018 [6,50]. 

In Table 2 we provide case reports of AbE after RT in which immunotherapy was utilized. Our PubMed search identified 60 additional cases of AbE since 2018. Most of these cases (14 cases) were observed in renal cell carcinoma (RCC). Ten cases occur in lung cancer, eight of them in non-small cell lung cancer (NSCLC). 

All reported cases had received prior treatments such as chemotherapy or resection surgery of the primary site or metastasis. Only a few patients had received RT previously. Immunotherapy was administered simultaneously with or immediately after RT. In these cases of AbE, the patients received markedly different RT dose schemes (8–70 Gy), fractionation sessions (1–35), and dose per fraction (1.8–28 Gy) with or immediately after additional immunotherapy. The vast majority of patients was treated with moderate hypofractionated (3–6 Gy/fr) or hypofractionated doses (>7 Gy/fr) at metastasis sites.

In fact, most AbEs have been reported in melanoma patients undergoing RT treatment along with immunotherapy (27 cases). Most patients received a total dose of 30 Gy (predominantly 3 Gy/fr), however, the RT schemes differed widely in doses (13.2–66 Gy) and number of fractionation sessions (1–52). Based on the number of AbEs reported after administration of checkpoint inhibitors, the blocking of the immune checkpoints seems to support AbE events.

## 3. Abscopal Effect Immune-Related Mechanisms

The precise mechanism of AbE remains unidentified, however, there are several independent and dependent mechanisms involved that are induced by certain radiation dose and fractionation schemes. 

RT is a highly effective anti-cancer therapy achieved by tumor cell lethality, prompted by irradiation-induced lethal DNA double-strand breaks (DSBs). Furthermore, the generation of free radicals also plays a role, as radiolysis of water in cells leads to production of reactive oxygen species (ROS) producing cascade of DNA damage in tumor microenvironment (TME) cells [101,102].

Recent advances in RT techniques allow for a more accurate delivery of higher doses per fraction [103]. Localized RT presents not only immunosuppressive but also immune-stimulatory effects, inducing the release of immune-stimulating tumor antigens via ICD [104]. Thus, it modulates the TME as a result of the induction of endothelial cell death and vascular damage as well as increased T cell priming in lymphoid tissues [105]. 

Demaria et al. revealed AbE to be dependent on the immune-mediated action of functional T cells. Hence, immune promotion is mainly supported by T cell priming against novel tumor-specific antigens [106]. Thereby, irradiated tumors can act as an in situ immunization, being functional T lymphocytes essential effector cells in such response [107]. Irradiation-modulated TME releases diverse cytokines and chemokines that recruit and mature antigen-presenting cells (APCs), namely dendritic cells (DCs) and macrophages to the tumor site. Decreasing tumor cells release elevated amounts of antigens, which are recognized and processed by APCs [108,109]. Tumor-related APCs prime antigen-specific effector T cells by presenting tumor peptide antigens to naïve T cells in tumor-draining lymph nodes (LNs) [110]. In fact, cytotoxic T lymphocytes (CTLs) recognize radiation-induced antigens and activated T cells that travel through the bloodstream to combat the residual tumor cells present in the non-irradiated distant metastatic sites which seems to lead to an effective anti-tumor immune response and cancer control [111]. 

Due to the stress response after the irradiation of the tumor cells, the damaged and dying cells release damage-associated molecular patterns (DAMPs) including heat shock protein 70 (Hsp70), calreticulin, adenosine-5′-triphosphate (ATP), and high mobility group protein B1 (HMGB1) [112]. Hsp70 has been seen to induce resistance against apoptosis when present inside the cells, however, extracellularly, it promotes apoptosis and contributes to an innate and adaptive immune response [113,114]. Calreticulin acts as an ‘eat me’ signal for DCs when exposed to the membrane resulting in enhanced phagocytosis of the cancer cells by DCs and, thus, results in an increased release of tumor-specific antigens (TSAs) [104,109,115]. Moreover, ATP binds to P2X purinoreceptor 7 (P2X7R) on inflammatory cells, activating the NOD-, LRR- and pyrin domain-containing protein 3 (NLRP3) inflammasome, a multiprotein complex that produces active caspase-1 and stimulates the production of interleukin (IL) 1β and IL-18 [116,117]. Il-1β mediates the antitumor-specific priming of the CD8+ T cells, IFN-γ production as well as the activation and maturation of DCs to APCs [118]. HMGB1 activates DCs, promoting their maturation and ATP release, leading to the enhancement of CD8+ effector T cells antigen presentation and their anti-tumor-specific activation [112,119].

Local RT induces the release of both pro- and antitumor cytokines due to irradiation-induced DNA damage. In the cytoplasm of irradiated cells, cyclic GMP-AMP synthase (cGAS) binds to translocated double-stranded DNA fragments (dsDNA) induced by RT and activates a signaling cascade via the second messenger 2′3′-cGAMP (cGAMP) and stimulator of interferon genes (STING) to produce type I interferons (IFNs) in the TME [120,121,122,123,124]. Radiation-induced interferons play a major role in the therapeutic effect of RT. In fact, irradiated tumor cells produce IFN-β and are, in turn, responsible for the release of IFN-γ by T cells [125,126]. IFN-γ consecutively promote cytotoxic T cell activation along with upregulating programmed cell death protein 1 (PD-1) receptor-ligand 1 (PD-L1) [127]. Following the irradiation-induced release of IFN-γ, there is an upregulation of pro-inflammatory chemokines CXCL10 and CXCL16 which are in turn responsible for the recruitment and activation of T cells into the TME [128,129,130]. ROS production leads to IL-1α, IL-1β and IL-6 production as well as tumor necrosis factor alpha (TNF-α) and transforming growth factor beta (TGF-β) [131]. 

Moreover, there are various mechanisms that can concomitantly suppress AbE such as those dependent on factors with immune system inhibitory effects. In particular, the environment of the irradiated volume plays an important role in this process. Thus, prevalence of immunosuppressive cytokines and cells present at the post-irradiated site could limit AbE regardless of immunotherapy treatment [132].

The hypoxic milieu within the irradiated tumor promotes recruitment of immunosuppressive regulatory T cells (Tregs) which induce immunosuppression in the TME [133,134]. Moreover, hypoxia impacts the antigen-presenting ability of APCs by reducing major histocompatibility complex (MHC) class I molecules. This reduction is correlated with a lower recognition of cytotoxic T cells and hence a reduced killing of tumor cells by these T cells [135]. 

In fact, a study correlated the frequency of AbE with the extent of adrenergic stress in a murine model. Enhanced norepinephrine production prevented T cell activation and consequently the abscopal response [136]. 

In most cases, RT alone is insufficient to overcome the established immune suppressive mechanisms of the TME. The immune effect of irradiation is affected by the applied dose, fractionation, and application volume making prediction of immunological reactions intricate [137]. RT in combination with immunotherapy may be advantageous in overcoming this immune tolerance and enhance AbE patient response. Preclinical studies have already illustrated how important the optimal RT dose, fractionation scheme, administration timing and dosing of the immunotherapeutics are to provide systemic antitumoral effects [106,138,139,140,141,142,143]. Identification of AbE prediction biomarkers may be crucial to develop an individualized medical approach. 

Recently, in the course of the ongoing Severe Acute Respiratory Syndrome Coronavirus 2 (SARS-CoV-2) pandemic, the effects of infectious diseases on the immune system have come into focus. The virus triggers inflammatory responses in the respiratory tract and induces a broad production of cytokines, which are also associated with the mechanism of AbE [123].

Herrscher et al. reported the first case of AbE in a SARS-CoV-2 infected patient with melanoma. The 84-year-old woman was diagnosed in early 2020 with peritoneal and a nodal recurrence of melanoma which was handled with palliative care. In late 2020, palliative RT was performed for a metastatic cervical node (20 Gy, 5 fractions). The patient was diagnosed with coronavirus disease 2019 (COVID-19) in early 2021. At this time point, metastases presented a shrinkage of 20–25% which may suggest an abscopal response linked to the activation of the innate immune system due to the SARS-CoV-2 infection and their influence on the production of pro-inflammatory cytokines [144]. 

Spontaneous tumor regression has been described over the last centuries being often correlated with acute bacterial, fungal, viral, or protozoal infections as well as vaccine therapy. In 25–80% of documented cases of spontaneous regression of cancer, the acute infections cause a strong immunological reaction presenting with fever, which in turn supports the regression of the tumor [145,146]. Thus, the increase in the anti-tumor response may also be responsible for the noted AbE after COVID-19 infection.

## 4. Abscopal Effect in Different Tumor Entities

AbEs have been reported in a variety of cancer types. In studies where additional immune therapy was absent, AbE occurred remarkably in cases of adenocarcinoma of different origins. Nevertheless, AbE were observed more frequently in melanoma patients also treated with immunotherapy. In breast and prostate cancer patients, the preclinical evidence for AbE cases is more robust compared to the frequency of reported clinical cases. Table 3 provides an overview of the frequencies of case reports of ABE in various tumor entities juxtaposed with their worldwide incidence and mortality.

### 4.1. Adenocarcinoma

Adenocarcinomas are malignancies arising from the epithelial cells of the glandular tissue in multiple sites of the body [148]. They usually originate in organs such as the gastrointestinal tract, esophagus, lung, breast, prostate or pancreas. However, in the case of metastasis, the determination of the primary site of origin is complex. In fact, adenocarcinomas account for 70% of cancers with unknown origin [149]. Twelve cases of AbE were observed under conventional treatment of adenocarcinomas, and 13 additional cases occurred after additional immunotherapy. 

Ehlers et al. reported the first clinical case of AbE in papillary adenocarcinoma of unknown origin. The 37-year-old female patient showed metastases in the bilateral neck, right axilla, and mediastinum LN. The bilateral neck and supraclavicular regions were treated with a total dose of 40 Gy in 20 fractions. After two weeks, the non-irradiated right axilla and mediastinum mass regressed, presenting the treated neck node only a partial response [8]. 

Adenocarcinoma AbE case reports of a digestive origin are underrepresented (three reports in total) considering that adenocarcinoma accounts for 95% of colon and rectal cancers [150]. In fact, Chuang et al. described a case of AbE after whole-brain irradiation in a female patient with metastatic adenocarcinoma of colorectal origin. Along with multiple brain metastases, the patient presented a huge metastatic lung mass. Treatment consisted of 30 Gy in 10 fractions without any adjuvant systemic therapy. The mass in the left lung markedly regressed two months later. These results suggest that AbE inducing factors are able to trespass the blood-brain barrier (BBB) [37]. Bonilla et al. reported a case of a patient with unresectable advanced gastric adenocarcinoma with extensive retroperitoneal LN involvement. The gastric mass and margins were treated with RT (30 Gy total), observing three months later a complete response of the non-irradiated retroperitoneal paraaortic adenopathy and gastro-hepatic ligament along with the tumor markers angiotensin-converting enzyme (ACE), carbohydrate antigen 19-9 (CA19-9) and cancer antigen 125 (CA-125) [41]. Furthermore, another AbE case was described in an adenocarcinoma patient undergoing RT (48 Gy; 24 fractions) at the primary tumor site for recurrent disease, along with adoptive T-cell immunotherapy and DC therapy. Two months after RT the metastatic lesion size had decreased in addition to serum tumor marker CA19-9 [60].

AbE have also been investigated in colon adenocarcinoma preclinical murine models. In fact, Baba et al. revealed an increased AbE in C57BL/6 mice related to the irradiated tumor volume and applied radiation dose, excluding anti-PD-1 immunotherapy [151].

Two cases of AbE in esophageal adenocarcinoma were reported until today. Esophageal adenocarcinoma starts in the mucus glands that line the lower part of the esophagus. When it metastasizes, the tumor cells accumulate in the LNs, lungs, liver, bones, adrenal glands, or brain [152]. Rees et al. reported AbE of all lung metastasis in a patient with lower esophageal adenocarcinoma. After irradiation of the primary esophageal tumor, lung metastases regressed for 14 months before cancer further progressed [13]. More recently, a patient treated with RT and esophagectomy for the primary tumor and associated LNs, presented signs of metastases regression twelve months following treatment [34].

### 4.2. Lung Cancer

Up to 85% of lung cancers are NSCLCs, with adenocarcinoma being the main subtype [153,154]. The pulmonary cancer cells spread typically to the contralateral lung, adrenal glands, bones, brain, or liver. Even if resection is the standard of care for early-stage NSCLC, RT is utilized as a first-line treatment for elderly, comorbid patients or as a palliative treatment in later stages [155,156,157]. In advanced or metastatic NSCLC, the combination of immunotherapy and RT significantly improves 1- and 3-year overall survival; as well as progression-free survival, though but is also beneficial in adenocarcinomas and PD-L1-negative patients [158].

Rees et al. reported the first AbE in a patient with metastatic lung adenocarcinoma treated with palliative radiation to the left lung and the mediastinum. After two weeks, the patient showed a regression in subcutaneous metastases in the forehead and the left shoulder [13]. Ever since there have been 17 more case reports of AbE in NSCLCs, respectively eight in lung adenocarcinoma. Five AbE cases resulted from irradiation of malignant tissue without support from any immunotherapy [24,36,43], wherein two of these reported cases described AbE as a result of the irradiation of brain metastases [27,38]. 

One case of NSCLC in the right upper lung lobe and metastases in the brain, left adrenal gland, left lower lung lobe, and the liver was treated with chemotherapy and brain RT. One month after RT, the adrenal, lung and liver lesions had diminished [27]. Furthermore, another patient with brain metastasis was treated with RT (25 Gy, five fractions) resulting in the regression of the primary tumor as well as a metastatic lesion in the left mediastinum one month following treatment [38]. 

A more recent study described an adenocarcinoma case with an origin in the right upper lung lobe. Three months post irradiation, the patient presented regression of the primary tumor and several non-irradiated LN. Serum carcinoembryonic antigen (CEA) levels were decreased in correlation with AbE, whereas CD8+ lymphocyte counts were low at the time the AbE was documented yet increased with metastases growth [43]. 

Between 2012 and 2021, twelve AbE cases were reported in pulmonary adenocarcinoma after treatment with RT and immunotherapy. Thereby, the AbE seems to occur after irradiation of the contralateral lung, the brain, liver and metastatic LNs [52,58,59,64,74,87,98]. In fact, four cases were described where patients received RT for their brain metastasis (from two weeks to four months after) with a resulting abscopal regression of the primary lung tumor after receiving anti-PD-L1 (atezolizumab) or anti-PD-1 (pembrolizumab) therapy [77,81,98].

### 4.3. Kidney Cancer

Renal cell carcinoma represents 3–5% of all new adult malignancies. Localized RCC is normally treated first line by resection with adjuvant immunotherapy or receptor tyrosine kinase (RTK) inhibitor therapy [159,160]. Stereotactic ablative radiotherapy (SABR) is recommended as treatment option for medically inoperable patients, though RT plays mainly a role as a palliative therapeutic option for relieving the symptoms of metastatic lesions [159].

The first AbE in renal cell carcinoma was reported in 1981 by Fairlamb. The 73-year-old female patient was diagnosed with lung, hila, and pubic bone metastasis next to her primary left kidney tumor. After nephrectomy, she was treated with palliative radiation to the groin (40 Gy, 15 fractions) which resulted in regression of the lung lesions two months after RT and remained disease-free for 4.5 years [10]. Since then five AbEs have been observed in RCC without adjuvant immunotherapy, while 18 AbE cases have been reported with predominantly nivolumab (anti-PD-1) as immunotherapy. In 1994, MacManus et al. identified a RCC (right kidney) patient presenting lung and mediastinal metastases. The patient received palliative radiation (20 Gy, 10 fractions) for pain relief, and after six months the paratracheal lymphadenopathy was reduced and lung metastases were non-detectable, suggesting an AbE. Cytokine assays (TNF-α, TNF-β, interferon gamma (IFN-γ), IL-2 receptor, IL-6) performed on a single serum sample during the regression of the metastases did not reveal any significant differences compared to the reference values [14]. 

Moreover, AbE was detected following irradiation of brain metastasis in a patient that underwent nephrectomy. Computed tomography (CT) examination leads to the identification of multiple metastases in bone, brain, lung, and mediastinum. The patient was treated with stereotactic radiosurgery to a dose of 18 Gy. Additionally, the patients vertebral bone lesion was irradiated with 40 Gy in five fractions. Treatment resulted in the regression of lung and mediastinal disease. Follow-up CT, one month after RT, revealed slight regression of the untreated multiple lung metastases and lymphadenopathy. However, three months following RT the patient developed new brain lesions, suggesting AbE to be organ-dependent and incapable of crossing the BBB [22]. Another report described the case of an RCC patient with multiple mediastinal, retroperitoneal, and cervical LN metastasis. The subject was treated after nephrectomy with the tyrosine kinase inhibitors (sunitinib) followed by a combination of anti-PD-1 antibody (AB) pembrolizumab and stereotactic body radiation therapy (SBRT) (32 Gy, four fractions). The patient achieved a systemic complete response within two months [63]. 

Most recently, Wong et al. identified nine cases of AbEs in the patient cohort of a descriptive and retrospective study of RCC. Approximately 20% of all observed patients revealed a regression in a non-irradiated tumor. The patients received besides RT either a single-agent anti-PD-1 AB (nivolumab, pembrolizumab) or a combination of anti-CTLA-4 and anti-PD-1 treatment (ipilimumab-nivolumab). RT was delivered in different radiation schemes depending on treatment indication and the location of the irradiated volume. AbE occurred in 26.7% of patients treated with combined immunotherapy and 16.1% of patients treated with a single agent. In addition, the abscopal response patterns were very divergent between the patients. Most patients experienced isolated abscopal responses in one to two lesions, while two patients presented a more extensive response in lung and liver [100].

### 4.4. Melanoma

The most effective treatment modality for localized melanoma is surgery, but RT plays a prominent role in the management of the disease. ICB is established as a standard of care for advanced melanoma [161]. With the employment of immunotherapy (pembrolizumab and nivolumab) for melanoma treatment in 30–40% of the patients a complete response was observed [162,163]. Adjuvant RT following lymphadenectomy and systemic therapy prevents local and regional recurrence. Thus, RT is highly effective and widely used as a palliative treatment being mostly utilized in brain, lung, liver or bone metastases.

Preclinical studies have investigated the influence of PD-1 expression on the systemic antitumor response induced by RT. A combination of RT in addition to PD 1 blockade results in the complete regression of the irradiated primary tumor as well as a reduction in size of non-irradiated secondary tumors outside of the radiation field. The tumors present infiltration of activated, cytotoxic CD8+ T cells, even in the non-irradiated tumors [164,165]. 

Since Postow et al. first discovered a correlation between AbE and immune checkpoint inhibitors, 26 other cases of AbEs associated with immunotherapy in melanoma patients have been observed, being the cancer type with the most reported abscopal immune responses [49].

One melanoma patient with melanoma of an unknown primary showed an AbE after conventional chemotherapy and palliative radiotherapy (total dose 20 Gy) for bilateral inguinal LNs. The other metastases were found to have gradually decreased in size, as seen on CT scan [35]. 

Different studies aiming to determine the occurrence rate for AbE in melanoma cases handled with RT combined with ICB. In a prospective clinical trial in which the combination of RT and systemic immunotherapy (ipilimumab) for melanoma was tested, the occurrence of an AbE was detected in 3 out of 16 patients (18.75%) (NCT01449279) [166]. Similarly, in another study 20–21% of melanoma patients who received RT in addition to ipilimumab exhibited an AbE following treatment [167]. Moreover, eleven cases of AbE were detected in a retrospective analysis of 21 patients with advanced melanoma which progressed after receiving ipilimumab and were subsequently treated with local RT. The median overall survival was extended to 22.4 months as a result of the AbE [54]. Another study reported two oligometastatic melanoma patients treated with anti-PD-1 and SBRT. One patient was diagnosed with BRAF-wild-type melanoma on the upper left leg with three liver metastases, a muscle lesion and a lesion in the left groin. The treatment consisted of RT to the three liver lesions along with adjuvant ICB (nivolumab). The LN metastases presented with a strong exhaustion signature defined by the markers PD-1, PD-L1, T cell immunoglobulin and mucin-domain containing-3 (TIM-3), lymphocyte-activation gene 3 (LAG-3), and the transcription factor thymocyte selection-associated high mobility group box protein (TOX). Moreover, a high level of the T cell-attracting chemokines CXCL9, CXCL10, CCL5, and CXCL13 were discovered. The patient rapidly developed a complete tumor regression, ongoing for more than 4.5 years. The other patient, however, was described with an unfavorable pre-treatment immune signature of the tumor mass without evidence of infiltration by exhausted T cells, the targets of anti-PD-1 treatment. The patient presented a primary melanoma in the right chest region followed by a single liver metastasis which were both surgically resected. Thereafter, more recent detected liver metastases were treated with RT (45 Gy, three fractions). A second SBRT was delivered displaying a month later, a complete regression of all metastases, lasting for over 2.5 years [97]. 

### 4.5. Breast Cancer

Breast cancer is the most commonly diagnosed cancer with an estimated 2.3 million new cases in 2020 [147]. RT is an effective tool for local tumor control, and decreases the risk of tumor cells dispersing within breast, chest area or axillary LNs. RT is also utilized as palliative treatment of symptomatic lesions in patients with metastatic breast cancer. RT is used to treat limited metastatic sites in only patients for whom surgical intervention is not an option (location of the metastases, general health state). 

In fact, a study described an AbE in a patient with metastatic breast cancer, after receiving RT alone. The patient presented with a 10 cm tumor in the right breast and multiple associated metastases in lung, bones and LNs, and was treated with RT alone as a palliative treatment, due to her performance status score of 3–4 in the Eastern Cooperative Oncology Group (ECOG). The applied radiation dose was distributed into 60 Gy to the right breast, 28 Gy to the left femur and 39 Gy to the lumbar vertebrae and sacrum. Following 10 months of RT, the lesions in the lung and LNs showed a prominent regression with normalized serum levels of CEA and cancer antigen 15-3 (CA15-3) [33]. Another report described an AbE in stage IV breast carcinoma after multiple fractions of high dose radiation [39].

The combination of RT and immunotherapy was also tested in metastatic breast cancer in clinical trials. Formenti et al. reported the results of a clinical trial investigating the different doses of a TGF-β inhibitor with RT (NCT01401062). Twenty-three patients with at least three distinct metastatic sites were randomized in two treatment arms. As a result, one patient with triple-negative breast cancer (TNBC), invasive breast cancer with limited treatment options and worse prognosis, showed a 28% reduction in the non-irradiated lesions. Unfortunately, no more details of this case were reported [168]. A separate study regarding patients with TNBC, performed post-mastectomy RT and reported a beneficial impact on 3-year breast cancer-specific survival which was dependent on the pathological stage of the patients [169]. 

Furthermore, preclinical models of metastatic breast cancer revealed a significant survival advantage when RT is combined with immune checkpoint inhibitors [138,139,170]. In fact, a study with a metastatic breast cancer murine model (TS/A) conducted irradiation of one of the tumors with different RT regimes in combination with an anti-CTLA-4 AB. Fractionated RT in combination with the AB leads to significant regression of the secondary tumor, nonetheless, RT alone did not induce AbE in this model [139]. In another murine breast tumor model (4T1) study with bilateral tumors, the non-irradiated tumor was growth restricted by RT and combined with anti-PD-1 AB resulting in prolonged survival, in addition to a reduction in spontaneous lung metastases. These results suggest that an effective modification of the immune microenvironment of non-irradiated tumor is generated by means of combination therapy [170]. 

Krombach et al. employed TNBC cells to examine the initial steps of RT-induced anti-tumor priming which is crucial for AbE advent, observing the strongest effects with high single doses [171]. In addition, in another murine TNBC model, RT effect was enhanced due to AbEs by immune checkpoint inhibitors and hyperthermia (HT). After implanting 4T1-Luc cells into the mammary glands of BALB/c mice, the primary tumors of the animals received RT (24 Gy, three fractions), HT, anti-PD-1 and anti-CTLA-4 immunotherapy. Results revealed a moderate reduction in both the primary tumors and the lung metastasis but improvement with respect to survival was observed [172]. 

### 4.6. Prostate Cancer

Prostate cancer is the second most frequently diagnosed cancer with 7.3% of all estimated cases in males worldwide [147,173]. The most aggressive form of this cancer type is defined by disease progression despite androgen deprivation therapy, carrying a poor prognosis with a median survival of less than three years [174]. The so-called castration-resistant prostate cancer (CRPC) is associated with a variety of symptoms such as bone metastases, spinal cord compression and advanced pelvic disease. The bone metastases are often radiosensitive and treated with palliative RT for pain relief [175,176].

The therapeutic response improvement in prostate cancer of RT in combination with immunotherapy has also been tested in clinical trials. In a phase I/II study, patients with metastatic CRPC received ipilimumab (anti-CTLA-4) as a monotherapy or in combination with RT. Patients treated with 10 mg/kg ipilimumab and RT showed the strongest response, however, no AbE was observed [177]. Furthermore, similar results were observed in a multicenter phase III trial with 799 patients, where a combination of ipilimumab and RT was used to treat bone metastatic castration-resistant prostate cancer (8 Gy), unable to induce AbE [178]. In a DC vaccine clinical trial an AbE was detected in a patient with advanced CRPC. In addition to the prostate mass, the patient showed metastases in the lungs, bone marrow and mediastinal and inguinal LNs. Six months following several cycles of DC vaccine with an immunostimulant and irradiation of the prostatic tumor as well as the inguinal LNs (24 Gy, three fractions), the size of mediastinal and retroperitoneal lesions was significantly reduced. Prostate-specific antigen (PSA) serum concentration post RT was decreased whereas CD3+ and CD8+ T cells as well as IL-6 serum concentration were slightly enhanced. All other assessed parameters were within range [67].

In prostate cancer, the incidence of an AbE is reduced by a characteristic suppression of the anti-tumor immune response as a reaction to RT. The immune response can be hindered by the recruitment of myeloid cells with immunosuppressive function to irradiated TME. A study determined that after primary tumor site irradiation, expression of macrophage colony-stimulating factor 1 (CSF1) increased significantly both in patients and preclinical prostate models. CSF1 enhanced recruitment of immunosuppressive myeloid-derived suppressor cells (MDSCs), thereby limiting the therapeutic response to RT and suppressing the immune-related mechanism of AbE [179].

AbE prospect in prostate cancer has been demonstrated in the preclinical stage. In fact, a CRPC preclinical model observed an increased median overall survival and AbE with combination of RT and immune checkpoint inhibition. Mice’s leg tumors were irradiated with 20 Gy, and additionally treated with anti-PD-1 or anti-PD-L1 ABs. Thus, RT leads to a local response in the irradiated tumor followed by a similar regression of the non-irradiated tumor graft in this model. This response was mediated by CD8+ T cells [180]. 

## 5. Description of Hypothetical AbE Patient

Based on the reported cases, the profile of a patient who is most likely to develop an AbE was created as a summary of the current data situation. 

The patient who is most likely to develop an AbE is a man aged between 42.4 and 72.6 years suffering from metastatic melanoma cancer. His primary tumor and also most likely this sentinel lymph nodes will be resected before the metastatic status is treated with systemic treatments (most probably ICB). Then, some of his metastases will be treated additionally with RT applied as a total dose of 30 Gy in 10 fractions. It will most likely be a brain metastasis that will be irradiated and the patient will receive at least four cycles of anti-PD-1 immunotherapy. This will most probably be 3 mg per kg body weight nivolumab every three weeks, and also the same dose of pembrolizumab. Around three months after this treatment, the irradiation of the brain metastases is most likely to result in the regression of non-irradiated lesions of the lungs followed by malignant LNs and liver lesions. However, a complete response could also be possible as a treatment result. 

Previously reported cases of AbE were predominantly observed in male patients under immunotherapy whereas the numbers of case reports of female patients were nearly the same with or without ICB. The mean age mentioned in the case reports was 57.5 years with a standard deviation of 15.1 years. The number of reports indicates that the highest probability for an AbE is the strongest after combined RT and immunotherapy in melanoma (27 cases), followed by RCC (18 cases) and then adenocarcinomas of lung origin (12 cases). In the most reported AbE cases, the primary tumor was resected (31 cases), systemic chemotherapy (19 cases) is indicated as the second primary treatment followed by RT (seven cases) as the third treatment procedure. In most AbE cases, the RT is applied as palliative treatment and most patients received a total dose of 30 Gy applied in 10 fractions. In melanoma cases, more than half of the AbEs occurred after irradiation of brain metastasis. As the most commonly used ICB agent, nivolumab was applied in a total of 39 cases of AbE followed by 17 cases previously treated with pembrolizumab. With a spread of 3.1 months, an average of 3.2 months elapsed between the applied RT and the abscopal response. The combination of RT and ICB were correlated in 37 cases with the abscopal regression of lung metastases, in at least 17 cases with the regression of malignant LNs and in ten cases where the non-irradiated liver lesions responded. A total response was described in 11 patients who showed an abscopal response (Table 2).

With the available data, no specific size or localization of the primary tumor, metastases nor radiation field could be defined that would enable an AbE to be predicted. Most reported clinical cases do not provide detailed information regarding blood parameters or other molecular tumor markers. 

Due to the relation between the appearance of an AbE and the anti-tumor immune reaction of the patient, the specific immunological pattern of patients would contribute significantly to the prediction of such a response. In particular, the change in the status of CD4+ and CD8+ T cells and the ratio of these cell groups to immunosuppressive Tregs or MDSCs during the entire treatment would be of particular interest. Unfortunately, only ten case reports in total provide details on this matter. When administering an immunotherapeutic agent, the determination of the PD-L1 status should be determined routinely. In 16 cases of AbE, this biomarker not only provided information for the benefit of an ICB but it also correlated with an abscopal response. In addition, the AbEs we have seen so far identify further markers that may potentially provide a pattern for predicting such an immunologically-driven effect. 

## 6. Potential Predictors for Abscopal Effect

It remains unclear whether an abscopal response can be predicted. AbEs occur unexpectedly after the irradiated tumors begin to grow following RT, as some cases may have gone clinically undetected or discovered by chance during routine examinations [50]. Therefore, predictive biomarkers would promote a better monitoring and investigation of this phenomenon. 

The T cell trafficking model was discussed as a predictive tool. In fact, Poleszczuk et al. developed a mathematical model to estimate the distribution of activated T cells between metastatic lesions. Thereby, they proposed that the migration of activated T cells from the activation site (irradiation field) to a specific metastasis depended on the physiologic blood flow to the organ involved. They aimed to identify treatment targets with the highest probability for AbE occurrence. They revealed that not all metastatic sites respond to the systemic immune response in an equal manner and that the dissemination of activated T cells among multiple metastatic sites is intricate. Consequently, the abscopal responses of the virtual patients presented the same metastatic sites, being highly patient-specific and could not be predicted from the data of previous cases [181]. Nevertheless, their model has some clinical limitations such as the gradual process of T cell priming and the influence of immune enhancement or suppression [182]. 

The current data situation only allows the application of a mathematical model to a small part of the mechanism behind an AbE, thus potentially misleading the clinical trial design. Therefore, timely identification of biomarkers associated with an abscopal response is necessary for predictive purposes. Even though some AbE biomarkers have already been discussed in literature no precise biomarkers have yet been validated to accurately predict AbE, especially in the context of combined treatment regimens. Thus, it is also worth considering immunotherapy predictive biomarkers for cancer immunotherapy and immune checkpoint inhibitors. Potential predictors for systemic responses to RT and immunotherapy include cytokine profiles as well as changes in AB titer and peripheral blood and/or tumor-infiltrating immune cells as listed in Table 4.

### 6.1. Tumor Protein p53

The tumor suppressor gene p53 regulates the expression of crucial proteins responsible for tumor cell proliferation, apoptosis, and DNA repair. The gene possesses a checkpoint function critical for preventing the replication of damaged DNA, thereby hindering thereby the growth of tumors, a function that is inhibited in mutated p53 [190]. Altered gene p53 functions are predominant driver mutations in numerous carcinomas.

Various preclinical studies have demonstrated p53 being involved in the mechanism behind AbE suggesting that p53 status could help predict the possibility of its occurrence. 

Camphausen et al. examined the dependence of AbE on the function of p53 after RT. They irradiated the normal tissue of non-tumor-bearing legs of wild-type (wt) p53 mice and p53-null mice to induce abscopal response in distant lung carcinomas and fibrosarcomas. Only the tumors in mice with a non-inhibited p53 protein complex showed markedly reduced growth rate after irradiation. These results may suggest that intact p53 could be necessary to trigger the AbE tumor which is independent yet radiation-dose dependent. Its transcription function could be essential for the expression of cytokines or to affect a systemic antiangiogenic effect resulting in an abscopal signal [183]. Furthermore, another study proved that non-irradiated tumors respond systematically to irradiation only if they express functional p53. Implanted human colon cancer cells in wt-p53 and p53-null mice were irradiated (dose of 10 or 20 Gy) delivered to only one flank tumor leaving the contralateral side non-irradiated. The irradiated tumors reacted dose-dependently but presented a tumor retardation independent of p53 status. A significant growth inhibition of non-irradiated tumors was observed exclusively in wt-p53 animals, displaying a higher effect at higher doses [184]. This preclinical data may suggest that p53-dependent signals might be responsible for AbE and that the evaluation of p53-status could help predict the possibility of an AbE, even a verification in a clinical environment is still pending.

### 6.2. Calreticulin

Calreticulin, as a calcium-binding endoplasmic reticulum protein, plays an essential function in antigen presentation ensuring the appropriate loading of cellular antigens [191]. Thus, calreticulin expression may also give some predictive information about a potential abscopal response to immunotherapy and radiotherapy [192]. Calreticulin is essential for the recognition of tumor cells by DCs and subsequently by T cells, in fact, depletion of calreticulin in the tumor cells reduced the T cell killing [193]. Sublethal irradiated tumor cells increase their calreticulin expression while radiation induces the translocation of calreticulin from the endoplasmic reticulum to the cell surface. The exposure on the cell surface, in turn, affects the induction of immunogenic cell death and enhances antigen processing which promotes the uptake of irradiated tumor cells by APCs [115,194,195]. Gameiro et al. examined the calreticulin expression in vitro on human breast, lung and prostate carcinoma cells in comparison to untreated control cells. Cells exposed to sublethal doses increased their susceptibility to CD8+ CTL-mediated lysis. Moreover, exogenous calreticulin also increased the CTL lyses of non-irradiated tumor cells regardless of their p53 or triple-negative phenotype [115].

### 6.3. Hsp70

The highly stress-inducible heat shock protein 70 (Hsp70) is overexpressed inside tumor cells in many different cancer types, being an indicator for enhanced cell growth, protection against lethal damage, resistance to radiation-induced cell death or shorter overall survival [196,197]. Hsp70 is known as a useful biomarker for the prediction of hepatocellular carcinoma, prostate cancer, serum squamous cell carcinoma (SCC) of the esophagus, endometrial carcinoma or node-negative breast carcinoma [198,199,200,201]. Moreover, Hsp70 is actively released by viable tumor cells within exosomes. The plasma levels of the Hsp70 have been linked to tumor burden in a mice model correlating levels with tumor progression as well as radiation-induced regression of the tumors [185]. Following RT, Hsp70 is emitted into the extracellular matrix by dying tumor cells in a smaller amounts than by viable tumor cells [202]. Herein, Hsp70 acts as a danger signal stimulating the innate and adaptive immune system [203]. Natural killer cells (NKs) are activated after binding to free Hsp70, which then migrate to Hsp70 positive tumor cells to eradicate them [204]. Furthermore, the free Hsp70 can bind tumor-derived antigenic peptides that stimulate T cells via antigen cross-presentation on MHC molecules of APCs [205]. Thus, free Hsp70 seems to be a potential biomarker for a radiation-induced anti-tumor immune response and hence, for AbE.

### 6.4. γ-H2AX

Phosphorylated histone H2AX (γ-H2AX) molecule amount measured after irradiation is an essential biomarker utilized to determine the DNA damage induced by radiation but may also give some information about AbE. In response to irradiation-induced DNA DSBs, cells phosphorylate thousands of H2AX molecules specifically at the damaged sites [206]. The amount of these y-H2AX foci is proportional to the radiation dose and can also be visualized in the chromatin of human peripheral blood lymphocytes (PBL) and hair follicles for use in biodosimetry [186,206]. Siva et al. observed the γ-H2AX foci in non-irradiated PBL and hair follicles of irradiated NSCLC patients to determine the abscopal response of the irradiation in normal tissue. They also correlated this to changes in plasma levels of CCL22 and CCL3 cytokines [186].

### 6.5. PD-L1

PD-L1 expression is the sole biomarker typically used in clinical practice for the prediction of immunotherapy responses. PD-1 is an immune checkpoint receptor expressed on activated T cells that regulate the T cell response antagonistically. The interaction of PD-1 and its ligand PD-L1, which is expressed on tumor cells and other cells in the TME affects T cell activation, resulting in an immune escape for cancer cells. The expression of PD-L1 on tumor-infiltrating lymphocytes must also be considered as a factor influencing its predictive value. Herbst et al. related the response to checkpoint inhibitors with increased levels of PD-L1 on the surface of the tumor-infiltrating lymphocytes but not on T cells [207]. 

Numerous studies have shown a correlation between a high PD-L1 expression and the response to immunotherapy [208,209,210,211]. PD-L1 expression alone has limitations in successfully predicting responses to immunotherapy, as it may also indicate T cell exhaustion and thus reduced systemic efficacy. Thus, it may imply either a limitation or complete absence of AbE [132,212]. 

Nevertheless, PD-L1 expression may deliver important information for an AbE event but not as a comprehensive and independent clinical biomarker. Several AbE reports correlated the expression of PD-L1 with patients’ systemic response [88,97]. Yaguchi et al. reported a PD-L1 positive immunohistochemistry (IHC) analysis in an AbE of a patient with recurrent pulmonary pleomorphic carcinoma and rapidly progressive metastases in the brain, bone, and pulmonary pleurae. The resected lung tumor strongly expressed PD-L1 and the non-irradiated metastases responsed as nearly complete after three cycles of anti-PD-1 immunotherapy [88]. Watanabe et al. analyzed PD-L1 status in two melanoma patients. One patient presented PD-L1 positive tumor lesions with a 40% chance of complete response due to the anti-PD-1 treatment alone. Hence, the authors questioned the role of RT in the outcome. The AbE in the other patient was correlated with the RT due to lack of the PD-L1 expression [97].

PD-L1 expression, may also serve to define the role of the RT in an AbE, however, the temporal and spatial heterogeneity of PD-L1 expression must also be taken into account [213]. Nonetheless, PD-L1 negative patients may also benefit from checkpoint therapy because PD-L1 expression in metastases do not correspond with the expression in the primary tumor [73,82,90]. A study of metastatic NSCLC demonstrated a substantial variation in the PD-L1 expression across different sites and clinical states. PD-L1 expression levels in the lung or distant metastatic biopsies correlated with a higher response rate, whilst no association could be observed in LN metastases [214]. 

### 6.6. Tumor Markers

Tumor markers could be used to monitor the response of the tumor masses; therefore, longitudinal marker measurement also allows detection of an ongoing AbE. 

Serum squamous cell carcinoma antigen (SCC Ag), a specific marker in SCC, could be associated with the outcome of RT. Elevated SCC Ag represents RT-resistant tumors whereas a reduction in its levels predicts a positive tumor response [215]. In the case of AbE, the SCC Ag levels also decrease due to irradiation and the following systemic response [31]. 

Similarly, the melanoma marker S100, commonly used as a marker for immunohistochemical identification of malignant melanoma, could help determine an abscopal response if measured longitudinally in short intervals. S100 levels were seen to correlate both with the tumor burden in distant metastases and the degree of infiltration of the liver and skeleton [216,217]. Immunotherapy combined with RT can lead to a decrease in S100 which could be associated with an AbE [97].

CEA is expressed as a non-specific tumor marker on the surface of many cancer cells, whereas CEA plasma levels are very faint (0 to 5 ng/mL). Increased CEA levels act as a determiner for the tumor stage and as a predictor of the response to therapy for gastric cancer [218,219,220]. In NSCLC, a CEA plasma level could be used as a prognostic marker for overall survival, recurrence or progression-free survival [221]. Additionally, a group analyzed CEA levels in a patient with metastatic NSCLC and reported an AbE following treatment, consistent with a drastic drop to normal levels in CEA levels during the systemic response [52]. Kuroda et al. also observed a significant drop in the plasma levels of a lung adenocarcinoma patient after RT and during the time of the occurrence of the AbE. CEA levels increased in accordance with the progression of a non-responder metastasis [43]. 

In breast cancer, high preoperative CA15-3 and CEA serum levels were described as directly related to tumor burden and outcome predictors [222,223]. In fact, Leung et al. reported a substantial decline CA15-3 levels in a breast cancer patient, along with a continuous decrease in CEA during the occurrence of the abscopal response [39]. 

Carbohydrate antigen 19-9 (CA19-9) is most commonly used as a tumor marker for pancreatic ductal adenocarcinoma [224], during an abscopal response this marker has seen to be reduced. Bonilla et al. reported a corresponding plasma concentration curve for a patient with gastric adenocarcinoma presenting an AbE following palliative RT [41]. Most recently, a case report of a patient with endometrioid adenocarcinoma reported decreasing plasma levels of CA125 during the time of the AbE event [48]. 

Unfortunately, most patients with AbE do not reflect the disease progress through specific tumor marker signatures [60,98].

### 6.7. ctDNA Amount and Tumor Mutational Burden

Circulating tumor DNA (ctDNA) are found in cell-free blood fraction and are described as a potent biomarker that enables the detection of tumor-specific sequence alterations [225,226,227]. Tissue tumor mutational burden (TMB) is a viable biomarker for the response to immunotherapy, helping to quantify tumor immunogenicity [228]. Elevated TMB supports the enhanced expression of tumor-associated neo-antigens following RT and subsequent anti-tumor immune response [229,230].

ctDNA abundance, the peripheral blood ratio between ctDNA and total circulating DNA, might also give information about tumor burden or disease progression. An independent predictor of the response to immunotherapy has been described as having a TMB higher than 16 when measured in ctDNA abundance [231]. ctDNA levels seem to be correlated to pseudo-progression in KRAS-mutated adenocarcinoma, a mutation associated with an improved outcome under immunotherapy. In pseudo-progressive patients, ctDNA levels decrease drastically whereas in progressive patients they appears strongly elevated [232]. 

Xie et al. measured the ratio between ctDNA and the total free DNA in the peripheral blood of an RCC patient which presented an AbE both before and during RT and pembrolizumab treatment. ctDNA abundance decreased before it unexpectedly rose two months after combination treatment and then drastically decreased again, suggesting its correlation with the extinction of tumor cells as corroborated by CT images [63]. Another study reported an AbE in a neuroendocrine cervical carcinoma patient with metastatic lesions in the liver, pelvic and retroperitoneal LNs. After performing a ctDNA test (70 genes), they found alterations in 19 genes indicating an elevated TMB. Tissue next-generation sequencing (NGS) also revealed a high TMB (53 mutations per megabase) [61]. 

Zhao et al. presented a case of an abscopal response in a patient with esophageal carcinoma and multiple LN metastases. Esophagectomy was performed followed by several cycles of chemotherapy, pembrolizumab and RT (42 Gy, six fractions) targeting the left retroperitoneal LN. After two months all LN metastases displayed complete regression. The ctDNA was analyzed to monitor the TP53 mutation. Results suggested that these mutations decreased significantly due to the applied combination therapy [72]. 

The changes in ctDNA release during or immediately after RT are a result of irradiation-induced immunogenic cell death. ctDNA presents a half-life of 0.5–2 h, therefore blood samples should be taken periodically and at shorter intervals to obtain more accurate information on the ctDNA alterations during patient treatment [233].

### 6.8. Tumor Antigens and Antibodies

RT generates tumor tissue damage leading to enhanced tumor-specific antigen release, which could function as an in situ immunization and induce AbE [107]. Consequently, serum analysis of tumor antigens alterations may be used as an abscopal response marker for RT alone and/or in combination with immunotherapy. 

The antigen load or more precisely the load of neoantigens, which are the number of mutations actually targeted by T cells, is associated with the response to immunotherapy [234,235]. Indeed, a study correlated a significantly increased neoantigen load in tumors of NSCLC patients after PD-1 blockade with a higher clinical response and improved progression-free survival. Thereby, the neoantigen-induced T cell response was detected after therapy initiation with its highest level three weeks following treatment starting with a progressive reduction [236]. AbE have been described to occur at different time points, suggesting that abscopal immune reactions might stimulate the priming of new T cells in addition to the initially primed one, originating a broader and more specific T cell response [182].

Measuring the concentration of specific antibodies against tumor-associated antigens may be illustrative. In effect, the serum of a melanoma patient was tested for auto-AB levels against melanoma antigen A3 (MAGEA3), revealing a systemic antitumor immune response. Following RT and immunotherapy the patient responded to cancer antigen PAS domain-containing 1 (PASD1) achieving complete remission [53]. Another research group reported an AbE of a melanoma patient treated with RT and ipilimumab. The cancer cells of the patient expressed the cancer antigen NY-ESO-1, an antigen found in 30–40% of advanced melanoma patients. The titers of AB against epitopes within the central portion of NY-ESO-1 in the serum samples collected during the treatment correlated positively with the disease progression. In fact, after RT and immunotherapy, the titers were increased by a factor of more than 30 corresponding to disease resolution [49]. 

Antigen immunogenicity, the ability of released antigens to elicit an anti-tumor immune response, is fundamental to the occurrence of abscopal responses and thus may be the limiting factor for an AbE [237]. Therefore, a further study promoted the determination of the tumor immunogenicity score (TIGS), represented as tumor antigenicity multiplied by antigen processing and presenting status, as an effective biomarker for immunotherapy response prediction [238]. TIGS may be best determined during the treatment period, on account of the relation between AbE and the immune response to tumor antigens.

### 6.9. Absolute Lymphocyte Count and Amount of Blood Immune Cells

The absolute lymphocyte count (ALC) and/or the count of specific immune cells could also act as conceivable predictive biomarkers for AbE. However, to date, only a few cases have given further details on the immune cell status of the respective patients during treatment.

In 1977, Antoniades et al. counted the absolute leukocytes as well as the subpopulations of neutrophils, monocytes, eosinophils, and basophils in their patients’ blood before and after irradiation compared with values of a healthy donor. The two patients with metastatic lymphoma exhibited a marked reduction in the size of non-treated abdominal LNs. Both patients showed an irradiation-induced decrease in the ALC corresponding with an increased ratio of neutrophils [9]. A more recent study analyzed leukocytes, neutrophils, lymphocytes, and monocytes in the blood of a patient with a diffuse-type giant cell tumor showing an AbE at one month after RT treatment of the right hilar in the left lung [28].

Diverse studies have shown that if more of these standard values were reported, changes in the immune cell amounts may be correlated with the occurrence of an AbE [43,49,52,54,187,188]. More precise information of the immune condition also enables an assessment of the immunosuppressive status that counteracts a possible AbE.

Golden et al. reported a case of AbE in a treatment-resistant lung cancer patient treated with RT and ipilimumab. Two to three months following RT to hepatic metastases, the non-irradiated lesions showed a decrease in size. Disease progression was tracked by ALCs, white blood cells (WBCs) and absolute eosinophil counts (AECs). Other studies associated an ALC increase of ≥ 1000/μL in whole blood with improved survival rates [239,240]. Indeed, the patient showed a correspondingly elevated ALC due to the RT and ipilimumab treatment combined with an AbE. The AEC was also enhanced by more than 100/μL whole blood, an increase which was previously also correlated with prolonged survival. Tumor-infiltrating lymphocytes (cytotoxic T cell, cytotoxic granules and regulatory T cells) also increased due to the treatment [52]. In a retrospective analysis of 21 advanced melanoma patients, an AbE was reported in 11 patients treated with a combination of ipilimumab and RT. In patients presenting AbE, ALC was increased prior to RT [54]. Another retrospective study analyzed 153 cases of patients with various cancer types treated with a combination of RT and immunotherapy, where at least one non-contiguous lesion was not irradiated. The results suggested a correlation between an enhanced post-RT ALC and abscopal response. Moreover, a lower post-RT APC results in a poorer progression-free and overall survival [188]. In brief, these data suggest ALC as a potential predictive AbE marker, however, this hypothesis requires additional data from clinical trials.

Due to their prominent role in AbE, different case reports also reported CD8+ lymphocytes values in blood at different treatment time points. There are reports of low CD8+ lymphocyte counts at the time of AbE, which increased again with disease progression [43]. A study measured the amounts of patients CD14+ MDSCs, CD8+ and CD4+ T cells as well as their activation status during the treatment period via flow cytometry. CD4+ ICOShigh T cells decreased during RT response while MDSCs first decreased severely before increasing again within the abscopal response period. The frequencies of IFN-γ-producing CD8+ and CD4+ T cells were decreased due to cell irradiation, yet increased slightly during AbE [49].

Additionally, the neutrophil-to-lymphocyte ratio may be an effective AbE predictive factor. In fact, eleven patients with different cancer entities presented an AbE with a combined RT and granulocyte-macrophage colony-stimulating factor (GM-CSF) treatment. A comparison was made between the baseline features of abscopal responders and non-responders. Accordingly, no significant differences were observed in phenotype parameters such as age, sex, previous numbers of RT or chemotherapy regimens nor in laboratory values (hemoglobin, albumin, WBC count) between responders and non-responders. However, a significantly lower baseline was noted in non-responders for the neutrophil-to-lymphocyte ratio [189].

### 6.10. Tumor-Infiltrating Lymphocytes

The evaluation of the tumor-infiltrating lymphocytes (TILs) may also give a potential indication of abscopal response. In preclinical studies, enhanced amounts of CD8+ cells in non-irradiated tumors were associated with the enhanced probabilities of AbE [151]. Clinically, the effectiveness of immunotherapy could be predicted as a result of TIL count determination [241].

Joe et al. analyzed the tissue of squamous carcinoma of the anal canal tumor with a complete response four months after palliative RT to the pelvic metastasis. Immunohistochemical staining for CD163, CD3, CD4, and CD8 showed heterogeneity of cell distribution. Some tumor regions were densely infiltrated with lymphocytes, including CD8+ and CD4+ T cells, suggesting a profuse immune response [31].

Moreover, a recent case report of a metastatic RCC patient reported a high heterogeneous infiltration of the non-irradiated primary lesion by CD8+ T cells. The patient showed regression of a lung metastasis six months after RT and immunotherapy treatment. Nevertheless, only a part of the tumor was considered immunogenic before anti-PD-1 treatment [90]. Teulings et al. performed a detailed immunological analysis of biopsies from a melanoma patient presenting AbE twelve months after irradiation of the axillary region and brain. Axillary LN sections exhibited a lymphocyte infiltration, whereas brain metastasis was mainly infiltrated by CD8+ T cells. Melanocyte-specific CD8+ T cells were found to represent an effector memory subgroup in both skin biopsies and blood. These data suggested a systemic and local anti-melanoma response against melanocyte differentiation antigens from the primary tumor and metastases [25]. Furthermore, the immunohistochemical analysis of the non-irradiated retropharyngeal metastasis of a patient with malignant pleural mesothelioma revealed a moderate infiltration of CD3+ cells after the occurrence of AbE. The intra-tumoral T cell population was composed of around 25% CD8+ T cells [99].

### 6.11. Cytokine Profiles

Radiation, as part of the cellular repair response, induces the release of cytokines and chemokines which in turn play various roles in systemic immune response modulation. Longitudinal observation of cytokine profiles could provide an overview about the systemic immune response. This information could provide some valuable therapy success predictive data in addition to assessing AbE incidence.

Animal models have already shown that increased levels of specific cytokines have a significant role in cytotoxic T cell responses and AbE [139]. In prostate cancer, several blood-based biomarker studies correlated elevated levels of different cytokines with disease progression and poor prognosis. After exposure to RT, the plasma levels of diverse cytokines, namely IL-1α, IL-4, IL-6, TNF-α, TGF-β, IFN-ɣ and macrophage colony stimulating factor (M-CSF) were increased and correlated with RT-induced toxicity [242,243,244,245]. In breast cancer studies, IL-6 and IL-8 were considered as potential markers for metastasis and disease progression [246,247,248]. Moreover, in patients with NSCLC undergoing palliative thoracic RT, significant changes in CCL15, CCL23, CCL24, CXCL2, CXCL6, CX3CL1, IL-6, and IL-8 serum levels were observed before, during, and after RT, correlating with metabolic tumor burden [249]. In hepatocellular carcinoma patients treated with RT, levels of IL-6 and IL-10 were seen to be valuable predictors of infield- and outfield-intrahepatic treatment failure [250]. Ohba et al. measured the serum concentrations of IL-1β, IL-2, IL-4, IL-6, TNF-α, and hepatocyte growth factor (HGF) in series before and after RT in a patient with hepatocellular carcinoma who presented AbE ten months after the irradiation of a thoracic vertebral metastasis. Most cytokine levels did not significantly change, except for TNF-α serum levels which had increased. The authors associated this increase with an enhanced NK cell activity along with increased AbE occurrence [15].

Immunosuppressive cytokines reduce the probability of AbE occurrence. Consequently, the balance between immune promoting and suppressing cytokines systemically and within the tumor environment before and after irradiation is crucial for treatment response. Levels of the cytokines TGF-β, IL-10 and adenosine should be particularly monitored. Radiation-induced cell death leads to the activation of M2 macrophages and TGF-β and IL-10 production within the irradiation field [251]. Moreover, Tregs are enriched in the irradiated tumor, releasing TGF-β and IL-10 [252]. In fact, increased Treg amounts together with radiation-induced recruitment of MDSCs exerts an impact on the production of immunosuppressive adenosine by irradiation stressed or injured cells [253]. On the other hand, IL-8, which primarily comes from circulating and intra-tumoral myeloid cells, inhibits the adaptive immune response by blocking the antitumor activity of effector T cells as well as antigen presentation [254,255]. Studies examining the clinical benefits of immune-checkpoint inhibitors linked high baseline levels of IL-8 in patients plasma to the poor prognosis of checkpoint inhibitor therapy. Consequently, a reduced immune response as a result of the IL-8 expression additionally suppresses the occurrence of an AbE.

### 6.12. Exosomes

Extracellular vesicles, including microvesicles and exosome, are small vesicles that contain bioactive cargo such as proteins, DNA or RNA, mediating intercellular communication in both physiological and pathological settings [256]. Exosomes secreted from tumor cells can carry both immune-stimulatory and immune-suppressive factors that partially mimic the profile of the releasing tumoral cells. Most previous studies have highlighted the immune-suppressive functions of these tumor-derived exosomes (TEXs). However, TEXs also transport immune-stimulatory TSAs as well as (co-)stimulatory immune molecules [257]. They play a crucial role in RT-associated immunity due to the antigen presentation and immune regulation dependent on the immune status of the TME [256]. TEXs have been studied to serve as liquid biopsy markers with high clinical diagnostic and prognostic value in lung, pancreatic, gastrointestinal cancer and hepatocellular carcinoma [258]. TEXs not only affect the local microenvironment around their releasing site but also circulate in the blood to distant metastatic sites promoting/suppressing tumor growth [257]. The immune-promoting effect is mainly based on the delivery of exosome-derived TSA to dendritic cells and on the activation of cytotoxic T cells to generate a specific anti-tumor response [259].

Following the irradiation production of TEXs containing DAMPs, chemokines and TSA are enhanced, however they vary according to the originally irradiated tissue [257,260,261]. The TEXs are processed by antigen-presenting cells, and trigger the activation of cytotoxic T cells which then migrate to distant non-targeted tumor cells and can induce AbE [257]. In case of AbE, Hsp70 surface-positive tumor-derived exosomes may be enriched in the irradiated field, facilitating their uptake by APC and thus triggering an enhanced anti-tumor response by NK cells [262,263].

In metastatic melanoma, TEXs could be used to predict the tumor response to anti-PD-L1 checkpoint inhibition. In this case, the level of circulating TEXs with surface PD-L1 could correlate with IFN-γ levels, which could distinguish responders from non-responders [264]. Additionally, exosomes may potentially be used to identify NSCLS patients who respond positively to immune therapy [265].

## 7. Clinical Studies on Abscopal Effect

Even after almost a decade of radioimmunotherapy and numerous studies on different immunotherapeutic agents, the occurrence of abscopal immune reactions has not been regularly achieved in clinical trials. The most frequently reported cases of AbE were correlated with the co-administration of RT and ICB, in particular with the administration of the anti-PD-1 therapy nivolumab. Nevertheless, a similar effectiveness has not yet been observed in clinical trials. Accordingly, trial design and evaluation must be performed taking into consideration many different aspects, namely, patient and endpoint selection, RT parameters, the particular definition of AbE, therapy sequence, among others, making the discovery of the optimal conditions extremely challenging [266].

A clinical trial tested the combination of RT with GM-CSF in patients with metastatic solid tumors of different origin (NCT02474186). The primary endpoint of the study was the proportion of patients with an abscopal response. Eleven of the fourty-one patients enrolled (26.8%) presented abscopal responses (four patients with metastatic NSCLC, 5, breast cancer patients and two, thymic cancer patients) [189]. A phase I study treated 20 stage IV melanoma patients with a combination of RT, nivolumab (anti-PD-1), and ipilimumab (anti-CTLA4), receiving immunotherapy shortly before and after palliative RT. Patients who received a conventional irradiation dose (3 Gy, 10 fractions) experienced in a 50% ratio an abscopal reaction extrinsic of the irradiation field whereas 11.1% of patients treated with hypofractionated irradiated scheme (9 Gy, three fractions) responded in a similar manner [267].

Contrary results were reported by a phase II trial examining the combination of RT and immune therapy in 20 patients with inoperable or metastatic melanoma (NCT02821182). The overall response rate (ORR) of the non-irradiated lesions increased by up to 45% with three complete and six partial responses similar to nivolumab monotherapy, however, no AbE was detected [268]. Subsequently, another study failed to observe an AbE in patients with metastatic head and neck squamous cell carcinoma (HNSCC) in a randomized, phase II trial of nivolumab combined with RT (NCT02684253). RT was delivered to one safely irradiated metastatic lesion between the first and second dose of nivolumab in three fractions adding up to a dose of 27 Gy. The patients in this treatment arm showed no significant prolonged survival compared to the immune monotherapy arm [269].

Current ongoing trials with AbE as their primary or secondary outcome mainly investigate the combination of RT and the inhibition of the PD-1/PD-L1 pathway in various cancers such as NSCLC, metastatic gastro-intestinal cancer, metastatic melanoma and metastatic breast cancer (Table 5). Several of them depict promising outcomes which may provide further insights into potential AbE predictive biomarkers.

In the ongoing clinical trial NCT03385226, among other objectives, the combination of RT (12 Gy, three fractions) and pembrolizumab is being observed over a period of 2 years for AbE rate. Immune cells will be functionally analyzed along with the diversity of T cell clones and tumor-infiltrating lymphocyte-specific neo-antigens and the expression of immunological checkpoints [270]. Moreover, another clinical trial aims to evaluate the treatment response at non-irradiated and extra-cranial sites of melanoma or NSCLC patients with brain metastasis, after receiving pembrolizumab along with stereotactic radiosurgery (6 Gy, 9 Gy, 18–21 Gy) (NCT02858869). As a secondary objective, potential immune biomarkers will be compared, analyzing blood samples for absolute cell counts, major lymphocyte populations as well as other serum immune biomarkers pre-, during and post-treatment for up to three years [271]. Another phase II study investigates the effect of pembrolizumab and palliative radiation therapy in patients with a metastatic esophagus, stomach or with gastroesophageal junction cancer (NCT02830594). Its primary endpoint aims to determine changes in tumor-infiltrating and circulating cytotoxic T cells, immunosuppressive Tregs and MDSCs at the metastatic examined sites. Cell populations will be correlated with the response rates of irradiated and non-irradiated tumors [272]. The benefit of radio-immunotherapy for advanced metastatic patients is being tested in a trial with a stratified patient cohort (NCT03453892). The systemic immune-modulating effect of the treatment will be assessed by deep immunophenotyping, evaluating up to 30 immune cell subtypes with their activation markers at various time points of the treatment [273]. Similar targets are being investigated in another trial (NCT03042156), which researches the effect of immune therapy and palliative RT combined treatment, along with inflammatory and irradiation-induced signatures as indicators for an AbE, particularly those extrinsic to irradiated sections [274].

## 8. Conclusions and Recommendations in the Frame-Work of 3P Medicine

AbE is a rare clinical phenomenon associated with RT, which, however, is becoming more frequent now towards improved radiation delivery and the development of immunotherapeutic drugs. The addition of ICB to conventional RT has led to an increase in the incidence of abscopal responses due to the involvement of various immune pathways in their occurrence. Nevertheless, the exact mechanism responsible for an AbE is still in question. It is observed after different treatment regimens and it takes weeks to months before an AbE becomes noticeable which makes it difficult to predict under typical clinical conditions.

The wide variation of the applied treatments often does not allow for a precise identification of the AbE’s origin, its triggers and contributors. Systemic therapies namely chemotherapy as well as immunotherapy may have delayed effects that later cannot be assigned to a specific treatment. To this end, the immune system of the patients can be affected by typical cancer pain management or immunosuppressive drugs such as corticosteroids. Furthermore, normal tissue in the radiation field also receives a small but still significant radiation dose during RT. Therefore, the observed AbE could also be related to low-dose radiation effects which are a result of the applied irradiation.

The costs of a combined therapy of ICB and RT has been estimated by Giuliani et al. to be 5866 EUR more expensive than immunotherapy alone, translates to 707 € to 1086 € per month of overall survival of NSCLC patients [275]. Targeted induction or an accurate prediction of an AbE by biomarkers could lead to a personalized, more precise and more cost-effective treatment strategy.

The reported clinical cases allow for the identification of several promising biomarker candidates which need to be further analyzed in the context of their predictive power. Validated biomarkers might be of great clinical utility

- to stratify patients
- to prognose treatment response individually by applying associated diagnostics
- to optimize treatment strategies tailored to every individual
- to create advanced multi-level diagnostic approaches based on the application of artificial intelligence (AI) for big-data analysis in the context of 3P medicine improving cost-efficacy of treatments and individual outcomes [276,277,278,279,280].

## Figures and Tables

**Table 1:** Abscopal effect case reports (no immunotherapy).

Reference	Age	Gender	Histo-pathology	Primary Tumor Site	Treatment of Orimary Tumor	Metastasis Site	Systemic Treatment	RT	Irradiation Site	Non-Irradiated Abscopal Regression	Time Frame for Abscopal Response	Reported Parameters
Lome et al. (1970) [7]	66	M	Transitional cell carcinoma of bladder	Bladder	RT	Lung, mediastinal + retroperitoneal LNs, adrenal glands	n/a	40 Gy, 4 weeks	Pelvis	Pulmonary lesions	5 months	Blood pressure, hematocrit, blood urea nitrogen,
Ehlers et al. (1973) [8]	35	F	Adeno-carcinoma (unknown origin)	Unknown	n/a	neck, axilla + mediastinum	n/a	40 Gy, 20 fr	Neck/supra-clavicular LN	Axilla and mediastinum	2 weeks	
Antoniades et al. (1977) [9]	44	M	Lymphoma	Left axillary + bilateral supra-clavicular LNs	RT	Right lung, abdominal LN	n/a	30 Gy, 20 fr	LN above diaphragm (mantle field)	Abdominal LN	n/a	Blood cunt values: hemoglobin, hematocrit, white blood cells (WBC), platelets, differential count of neutrophils, eosinophils, basophils, lymphocytes + monocytes
	40	M	Lympho-cytic lymphoma	Right axillary LN	RT	Abdominal LN	n/a	30 Gy, 20 fr	LN above diaphragm (mantle field)	Abdominal LN	n/a	
Fairlamb et al. (1981) [10]	73	F	Renal cell carcinoma (RCC)	Left kidney	Nephrec-tomy	Left groin, lung, hila, pubic bone	n/a	40 Gy, 15 fr	Groin	Lung	3 month	
Rees et al. (1981) [11]	n/a	n/a	Mixed-cellularity Hodgkin lymphoma	n/a	n/a	n/a	n/a	35 Gy, 28 days	Mantle field	Pelvis	n/a	
	n/a	n/a	Pre-dominant Hodgkin	n/a	n/a	n/a	n/a	36 Gy, 24 days	Mantle field	Pelvis, para-sortic nodes	n/a	
	n/a	n/a	Recurrenced Hodgkin unspecific	n/a	n/a	n/a	n/a	38 Gy, 39 days	Para-aortic and pelvic nodes	Left axilla LN	n/a	
	n/a	n/a	Mixed-cellularity Hodgkin lymphoma	n/a	n/a	n/a	n/a	40 Gy, 29 days	Mantle field	Para-aortic region	n/a	
	n/a	n/a	Brill Symmers disease	n/a	n/a	n/a	n/a	21 Gy, 15 days	Chest wall, right axillary	Left axilla, spleen	n/a	
	n/a	n/a	Lympho-sacroma	n/a	n/a	n/a	n/a	18 Gy, 13 days	Right groin	Right neck LN	2 month	
	n/a	n/a	Lymphoma	n/a	n/a	n/a	n/a	35/20 Gy, 34 days	Right breast, pelvis	Mediastinal mass	n/a	
	n/a	n/a	Reticulum cell sarcoma	n/a	n/a	n/a	n/a	39 Gy, 43 days	Para-aortic and pelvic nodes	Right axilla	n/a	
	n/a	n/a	Follicular lymphoma	n/a	n/a	n/a	n/a	30 Gy, 22 days	Neck, supra-clavicular area	Bilateral axilla	n/a	
Robin et al. (1981) [12]	59	F	Histiocytic lymphoma	Right kidney	COPP, BACOP	Left kidney	n/a	20 Gy, 10 fr	Right kidney	Left kidney	n/a	
Rees et al. (1983) [13]	49	M	Adeno-carcinoma (esophagus)	Esophagus	n/a	Lung	n/a	40 Gy, 20 fr	Esophagus	Lung	14 month	
	n/a	n/a	Adeno-carcinoma (lung)	Lung (left lower lobe)	n/a	Mediastinum, subcutaneous metastases (forehead, left shoulder)	n/a	35 Gy, 10 fr	Medi-astinum and left lower lung	Subcutaneous metastases (forehead, left shoulder)	2 weeks	
MacManus et al. (1994) [14]	58	M	Renal cell carcinoma (RCC)	Right kidney	RT	Left lung, paratracheal lymphadeno-pathy	Medroxy-proges-terone acetate	20 Gy, 10 fr	Right kidney	Lung, paratracheal LN	4 month	TNF-α, TNF-β, INF-γ, IL-2 receptor, IL-6
Ohba et al. (1998) [15]	76	M	Hepato-cellular carcinoma	Liver	Resection/CT	Second thoracic vertebra	Acetate	36 Gy, n/a	Thoracic vertebral	Liver	10 months	Hemoglobin, leucocyte, platelet count, bilirubin, aspartate amino-transferase, alanine amino-transferase, albumin, alkaline phosphatase, AFP, PIVKA-II, IL-1β, IL-2, IL-4, IL-6, HGF, TNF-α
Nam et al. (2005) [16]	65	M	Hepato-cellular carcinoma	Liver	n/a	Skull, ribs (3–6th), sternum	n/a	30 Gy,	Skull	Liver, ribs, sternum	10 months	AST, ALT, AFP
Wersäll et al. (2006) [17]	83	F	Renal cell carcinoma (RCC)	Right kidney	n/a	Lung, LN, abdomen	n/a	32 Gy, 4 fr	Kidney	LN, lung	2 years	
	55	F	Renal cell carcinoma (RCC)	Right kidney	n/a	LN, aorta, liver	n/a	32 Gy, 4 fr	Kidney	Lung	5 months	
Takaya et al. (2007) [18]	69	F	Uterine cervix	Pelvic	n/a	Para-aortic LN	n/a	28.8 Gy, 16 fr	Pelvis	Para-aortic LN	n/a	serum levels of squamous cell carcinoma (SCC) antigen
								22 Gy, 21 fr				
								24 Gy, 4 fr				
Isobe et al. (2009) [19]	65	M	Natural killer cell lymphoma	n/a	n/a	Skin, submandibular LN	Chemo-therapy	40 Gy	Skin	Sub-mandibular LN	2 months	WBC, haemoglobin, platelet count, serum lactate dehydrogenase, IL-2, TIA-1, granzyme b, CD2, CD3, CD4, CD5, CD7, CD8, CD16, CD19, CD20, CD25, CD30, CD38, CD56, TCR αβ, TCR γδ.
Lakshmanagowda et al. (2009) [20]	65	F	Chronic lymphocytic leukemia	Leukemia	n/a	Right axillary LN, right neck LN	n/a	24 Gy, 12 fr	Right axillary LN	Right neck LN	During treatment	
Okuma et al. (2011) [21]	63	M	Hepato-cellular carcinoma	Liver	n/a	Lung, mediastinal LN	n/a	60.75 Gy, 27 fr	Mediastinal LN	Lung	1 month	
Ishiyama et al. (2012) [22]	61	M	Renal cell carcinoma (RCC)	Left kidney	Nephrec-tomy	Left adrenal gland, lung, multiple mediastinal + hilar LN, bone, spine, brain	n/a n/a	18 Gy	Brain	Lung, LN	1 month	
								40 Gy, 5 fr	bone, spine			
Tubin et al. (2012) [23]	72	M	Medullary thyroid carcinoma	Thyroid	Thyroidec-tomy	Left supraclavicular LN, left infraclavicular LN, mediastinal LN levels 4R + 6	n/a	30 Gy, 3 fr	4R LN level	6 LN level	1 month	
Siva et al. (2013) [24]	78	F	Non-small-cell lung carcinoma (NSCLC)	Lung (left upper lobe + right lower lobe)	Carboplatin, paclitaxel, RT	Right adrenal, right humeral head	Chemo-therapy (carboplatin, paclitaxel)	60 Gy, 30 fr	Left upper lobe	Right adrenal, right humeral head	12 months	
								26 Gy, 1 fr	Right lower lobe			
Teulings et al. (2013) [25]	67	M	Melanoma	Left scapula	Resection	Axilla + suprascapular region (LN), brain	Chemo-therapy	50 Gy, 30 fr	Axilla + supra-scapular region	n/a	n/a	S100, CD8, CD68, CD3, IL-4, IL-10, Il-17, TNF-α, IFN-γ,
								20 Gy, 4 fr	Whole brain	Lung, mediastinum	2 weeks	
Lock et al. (2015) [26]	71	M	Hepato-cellular carcinoma	Liver	RT	Lung	n/a	70 Gy, 15 fr	Liver	Lung	4 months	AFP, liver enzymes
Yarchoan et al. (2015) [27]	60	M	Non-small-cell lung carcinoma (NSCLC)	Lung (right upper lobe)	n/a	Brain, hilar LN, left adrenal gland, left lower lung lobe, liver	Chemo-therapy	Unknown	Brain	Adrenal, lung, + liver	1 month	History of tobacco use, CK7, TTF-1, CK20, EGFR
Desar et al. (2016) [28]	19	M	Diffuse-type giant cell tumor	Knee	Imatinib, femoral amputation	Lung, mediastinal LN, right hilar	Tyrosine kinase receptor inhibitor (imatinib), steroid	30 Gy, 10 fr	Right hilar	Left lung	1 month	Hemoglobulin, leukocytes, platelets, neutrophils, lymphocytes, monocytes, sodium, albumin, CRP
Orton et al. (2016) [29]	84	M	Pleomorphic soft tissue sarcoma	Scalp	Resection	Pinna of left ear, parotid gland, lung	n/a	40 Gy, 8 fr	Post-auricular lesion	Lung	2 months	
Saba et al. (2016) [30]	69	F	Multiple myeloma	n/a	Chemo-therapy (melphalan, prednisone)	Left humerus, bilateral clavicles, right scapula left skull, right anterior + posterior thigh, stomach	Chemo-therapy (melphalan, prednisone)	150.5 Gy	Left humerus, bilateral clavicles, right scapula, left skull, right anterior LN + posterior thigh, stomach	Head of left triceps	5 months	Serum IgG levels
Joe et al. (2017) [31]	57	F	Squamous carcinoma of anal canal (SCCA)	Anal canal	RT	Mesorectum, perirectal, right internal iliac + obturator LN, liver, right liliac bone	Chemo-therapy	54 Gy, 30 fr	Primary anal tumor, affected pelvic lymph nodes, iliac bone metastasis	Liver	1 month	SCC antigen, PD-1, PD-L1, CD163, CD3, CD8 expression of tumor-infiltrating lymphocytes (TILs)
Lesueur et al. (2017) [32]	89	M	Neuroendocrine large-cell thymic carcinoma	Thymus with sternal extension	n/a	Lung, pancreas, right lower paratracheal LN	n/a	29.6 Gy, 8 fr	Sternum	Lung	4 months	CK7, synaptophysin + chromogranin A + negative for CK5, CK6, CK20, TTF-1, S100, PSA, melan-A
Azami et al. (2018) [33]	64	F	Breast carcinoma	Right breast	Irradiation	Lung, bone (femur, lumbar vertebrae + sacrum), LNs (lung, right axilla, right supraclavicular area, mediastinum)	n/aa	60 Gy	Right breast	Lung, LNs	10 months	HER2, KI-67, CEA, CA15-3
								28 Gy	Reft femur			
								39 Gy	Lumbar vertebrae + sacrum			
Bruton Joe et al. (2018) [34]	74	M	Adeno-carcinoma (esophagus)	Esophagus	Esophagec-tomy	LN, right renal vessels	n/a	30 Gy, 10 fr	Primary tumor, closest LN	Renal vein	12 months	Hemoglobin
Chantharasamee et al. (2018) [35]	51	F	Melanoma of unknown primary	Left lower extremities left inguinal mass	n/a	Bilateral inguinal LN, multiple LN throughout abdominal + pelvic cavity	Chemo-therapy (carboplatin, paclitaxel)	20 Gy, NR	Bilateral inguinal LN	LNs	6 months	Vimentin, S100, HMB-45
Chino et al. (2018) [36]	58	M	Non-small-cell lung carcinoma (NSCLC)	Lung	Surgery, chemotherapy	Liver	Chemo-therapy	60 Gy, 8 fr	Lung	Liver	5 months	AFP
Chuang et al. (2018) [37]	74	F	Adeno-carcinoma (colon)	Colorectal cancer	n/a	Left lung, liver, brain	n/a	30 Gy, 10 fr	Brain	Left lung	2 months	CK20, CDX2, thyroid transcription factor 1 (TTF-1), CK7
Hamilton et al. (2018) [38]	47	M	Non-small-cell lung carcinoma (NSCLC)	lung (left upper lobe)	n/a	Left mediastinum, bilateral hilar, brain	n/a	25 Gy, 5 fr	Brain	Left upper lobe left mediastinum	1 month	Previous illness
Leung et al. (2018) [39]	65	F	Ductal carcinoma	Right breast	n/a	8th thoracic vertebra, axillary LN	n/a	225 Gy, 15 fr	Breast	Axillary LN	12 months	Hemoglobin concentration, CEA, CA-125, CA15-3
								50 Gy, 25 fr	Thoracic bone			
Agyeman et al. (2019) [40]	56	M	Dermatofibro-sarcoma protuberans (DFSP)	Left lower leg	Local excision	Posterior torso	Chemo-therapy (imatinib mesylate)	40 Gy, 20 fr (Cobalt-60)	Left lower limb	Posterior torso	5 months	CD34, actin, desmin, S100
Bonilla et al. (2019) [41]	78	F	Adeno-carcinoma (gastric)	Gastric antrum	Gastrojejunostomy	Retroperitoneal space		30 Gy, 10 fr	Gastric mass + margin	Retro-peritoneal paraaortic adenopathies + gastrohepatic ligament	3 months	carbohydrate antigen 19-9 (CA 19-9), CA 125, ACE
Kim et al. (2019) [42]	70	M	Cholangiocarcinoma	Lung (right upper lobe)	Chemo-therapy	Liver	Chemo-therapy	48 Gy, 4 fr	Right upper lung lobe	Liver	3 months	Bilirubin, history of tobacco use, alcohol consumption, CK7, TTF-1, CA 19-9, Napsin A, CK20, anaplastic lymphoma kinase (ALK), PD-L1,
Kuroda et al. (2019) [43]	76	F	Adeno-carcinoma (lung)	Lung (right upper lobe	Right upper lobectomy	Multiple mediastinal + right hilar LNs	n/a	60 Gy, 30 fr	Multiple mediastinal + right hilar LNs	New left hilar + right supra-clavicular LN, lung	3 months	EGFR mutation, PD-L1 tumor proportion score, CEA, CD8+
Ellerin et al. (2020) [44]	84	F	Non-small cell carcinoma (right gland)	Deep lobe of right parotid gland	n/a	Left upper lobe, bilateral pulmonary masses, bilateral hilar, left paratracheal LNs in right upper lobe, right cervical LN, mediastinal LN	n/a	50 Gy, 20 fr	Primary site	Lung, mediastinal LN	2 weeks	History of tobacco use, previous illness, Lactate dehydrogenase, CK7, GATA-3, CK5/6, p40, PD-L1, mutations in NTRK1, NTRK2, NTRK3
Guan et al. (2020) [45]	76	F	B3 thymoma	Thymus	Palliative RT	Multiple lung/LN metastases	n/a	66 Gy, 33 fr	Not clarified	Regression of thymic lesion + lung metastases in non-irradiated area	2 months	PD-L1
Ohmatsu et al. (2020) [46]	77	M	Hepato-cellular carcinoma	Liver (right lobe)	hepatic segment-ectomy	Liver, inferior vena cava, lung	n/a	42 Gy (boost), 14 fr	Inferior vena cava	Lung	1 month	Mediacal history, AFP,
Mazzaschi et al. (2021) [47]	66	M	High-grade squamous cell carcinoma	Lateral pharyngeal wall	Chemo-therapy, RT	Multiple pathological left cervical LNs left humerus	Chemo-therapy (docetaxel, cisplatin, 5-fluorouracil)	70 Gy	Oropharynx, rhino-pharynx, hypo-pharynx, larynx, bilateral jugular-digastric LNs	Humerus	2 months	p53 hyper-expressed
								40 Gy	Cervical + V level LNs			
Tomita et al. (2021) [48]	88	F	Adeno-carcinoma	Uterus	Radical hysterectomy with bilateral salphingooph-erectomy	Lung, recurrent primary	n/a	65 Gy, 26 fr	Recurrent primary	Multiple lung metastases	5 months	Medical history, CA-125

Abbreviations: M: male; F: female; n/a: not available; RT: radiotherapy; LN: lymph nodes; WBC: white blood cells; fr: fractions; RCC: renal cell carcinoma; TNF-α: tumor necrosis factor alpha; IFN-γ: interferon gamma; IL: interleukin; CT: computed tomography; AFP: α fetoprotein; HGF: hepatocyte growth factor; AST: aspartate aminotransferase;ALT: alanine aminotransferase; SCC: squamous cell carcinoma; TIA-1: TIA1 Cytotoxic Granule Associated RNA Binding Protein; TCR: T-cell receptor; NSCLC: non-small-cell lung carcinoma; CK: cytokeratin; TTF-1: thyroid transcription factor 1; EGFR: epidermal growth factor receptor; CRP: C-reactive protein; SCCA: squamous carcinoma of anal canal; PD-1: programmed cell death protein 1; PD-L1: PD-1 receptor-ligand 1; TIL: tumor-infiltrating lymphocyte; PSA: Prostate-specific antigen; HER2: human epidermal growth factor receptor 2; CEA: carcinoembryonic antigen; CA15-3: cancer antigen 15-3; CA-125: cancer antigen 125; CA 19-9: carbohydrate antigen 19-9; ALK: anaplastic lymphoma kinase; DFSP: Dermatofibro-sarcoma protuberans.

**Table 2:** Abscopal effect case reports with immunotherapy.

Reference	Age	Gender	Histo-Pathology	Primary Tumor Site	Treatment of Primary Tumor	Metastasis Site	Systemic Treatment	RT	Irradiation Site	Non-Irradiated Abscopal Regression	Time Frame for Abscopal Response	Immuno-Therapy	Reported Parameters
Postow et al. (2012) [49]	33	F	Melanoma	Upper back	Excision	Lung, right hilar LN, paraspinal region, spleen	Chemo-therapy, immuno-therapy	28.5 Gy, 3 fr	Paraspinal region	Right hilar LN, spleen	4 months	Ipilimumab (CTLA-4)	BRAF mutation, NY-ESO-1 expression, CD4+ ICOS^high^, CD14+ HLA-DR^low^, CD8+, CD4+ T cells, seromic analysis
Hiniker et al. (2012) [51]	57	M	Melanoma	Left posterior arm	Excision	Left axillary, liver, left upper arm	Immuno-therapy	54 Gy, 3 fr	Liver	All metastases	6 months	Ipilimumab (CTLA-4)	
Golden et al. (2013) [52]	64	M	Adeno-carcinoma (lung)	Lung	Chemo-therapy (pemetrexed, carboplatin, gemcitabine, vinorelbine)	Left + right supra-clavicular LN, right upper lobe, left lower lobe, bilateral hilar, mediastinal adenopathy, liver, sacrum	Chemo-therapy (pemetrexed, carboplatin, gemcitabine, vinorelbine), immuno-therapy	59.4 Gy, 33 fr	Right lung, right supra-clavicular, right hilar, mediastinal adenopathy		2 months	Ipilimumab (CTLA-4)	Absolute lymphocyte counts (ALCs), absolute eosinophil counts (AECs), metabolic activity LN, CD8 cytotoxic T cells, TIA-1 (cytotoxic granules), FoxP3+ (Tregs), CK7, TTF-1, CK20, CDX2, CEA level
								30 Gy, 5 fr	Liver	Left lung, right lung, hilar adenopathy	3 months		
Stamell et al. (2013) [53]	67	M	Melanoma	Head	RT	Forehead, scalp, neck, nodal, brain	Chemo-therapy, immuno-therapy	24 Gy, 3 fr	Primary tumor	Skin metastasis	8 months		Melanoma antigen A3 (MEGA3), PAS domain containing 1 (PASD1) level of serum
								SRS	Brain	Complete remission		Ipilimumab (CTLA-4)	
Grimaldi et al. (2014) [54]	n/a	n/a	Melanoma	n/a	n/a	n/a	Immuno-therapy	30 Gy, 10 fr	Brain	Liver	n/a	Ipilimumab (CTLA-4)	
	n/a	n/a	Melanoma	n/a	n/a	n/a		30 Gy, 10 fr	Brain	Pelvic relapse	n/a		
	n/a	n/a	Melanoma	n/a	n/a	n/a		50 Gy, 25 fr	Chest wall, right axilla	Liver, cutaneous metastases	n/a		
	n/a	n/a	Melanoma	n/a	n/a	n/a		20 Gy, 5 fr	Right inguinal lymph node	Gastric, cutaneous, lung, lymph nodal + retroperitoneal abdominal metastases	n/a		
	n/a	n/a	Melanoma	n/a	n/a	n/a		30 Gy, 10 fr	Brain (WBRT)	Liver, bilateral axillary + right ovaric metastases	n/a		
	n/a	n/a	Melanoma	n/a	n/a	n/a		30 Gy, 10 fr	Brain (WBRT)	Lung, cutaneous, lymph nodal + abdominal metastases	n/a		
	n/a	n/a	Melanoma	n/a	n/a	n/a		30 Gy, 10 fr	Right chest wall	Lymph nodal, cutaneous + chest wall metastases	n/a		
	n/a	n/a	Melanoma	n/a	n/a	n/a		30 Gy, 10 fr	Vertebral metastasis	Lung metastases	n/a		
	n/a	n/a	Melanoma	n/a	n/a	n/a		24 Gy, 1 fr	Brain (SRT)	Cutaneous metastases	n/a		
	n/a	n/a	Melanoma	n/a	n/a	n/a		20 Gy, 1 fr	Brain (SRT)	Liver metastases	n/a		
	n/a	n/a	Melanoma	n/a	n/a	n/a		24 Gy, 1 fr	Brain (SRT)	Lung metastases	n/a		
Thallinger et al. (2014) [55]	44	M	Melanoma	n/a	n/a	Liver, lung, right kidney, right adrenal gland, LN, bone, brain	Chemo-therapy, immuno-therapy	30 Gy, 10 fr	Brain	Kidney, lunge, liver	2 months	Ipilimumab (CTLA-4)	S100, HMB45, Melan A, BRAF, NRAS + c-KIT mutations
Okwan-Duodu et al. (2015) [56]	50	F	Melanoma	Right lower back	Excision	Right groin, right occipital area, right suboccipital node, sub-centimeter right pulmonary node, brain, multiple retroperitoneal, subcutaneous, aortocaval, left periaortic + peripancreatic LNs	Immuno-therapy	Whole-brain RT	Brain	Pulmonary, retroperitoneal, mesenteric nodes.	5 months	IL-2 therapy	
Michot et al. (2016) [57]	33	M	Classical Hodgkin disease	Nodal supra-diaphrag-matic area	Chemo-therapy (doxorubicin (Adriamycin), bleomycin, vinblastine, dacarbazine)	Mediastinal right hilar + coeliac areas LN, supra-diaphragmatic + subdiaphragmatic LN, mediastinal lymphadenopathy	Chemo-therapy (doxorubicin (Adriamycin), bleomycin, vinblastine, dacarbazine), immuno-therapy	30 Gy, 10 fr	Right hilar mediastinal LN	n/a	n/a	Pembrolizumab (PD-1)	
Cong et al. (2017) [58]	64	F	Non-small-cell lung carcinoma (NSCLC)	Left lung	Chemo-therapy (cisplatin, pemetrexed)	Para-mediastinal tumor, lingual segment tumor	Chemo-therpay, immuno-therapy, EGFR inhibitor	37.5 Gy, 5 fr	Para-mediastinal tumor	Lung	10 months	Endritic cells + cytokine-induced killers (DC-CIK) immunotherapy	EGFR mutation
Komatsu et al. (2017) [59]	60	M	Adeno-carcinoma (lung)	Lung (right upper lobe)	Lobectomy	Liver metastasis, intrapulmonary metastasis	Chemo-radio-therapy, immuno-therapy	40 Gy, 20 fr	Liver	Lung	3 weeks	Nivolumab (PD-1)	
Sato et al. (2017) [60]	54	M	Adeno-carcinoma (gastric cancer)	Colon	Distal gastrectomy	Peritoneal tumor	Chemo-therapy (TS-1, paclitaxel), immun-otherapy	48 Gy, 24 fr	Colon	Peritoneal tumor	2 months	T-cell immuno-therapy, dendritic cell (DC) therapy	CEA, CA19-9
Sharabi et al. (2017) [61]	48	F	Neuro-endocrine cervical carcinoma	Uterine, cervix	Chemo-therapy, RT	Liver, pelvic, retro-peritoneal + pelvic LN	Chemo-therapy (cisplatin, etoposide), immuno-therapy	20 Gy, 4 fr	Abdominal mass	Hepatic lesion, pelvic mass, pelvic + retroperitoneal LN	4 month	Nivolumab (PD-1)	Pan-cytokeratin, synaptophysin, CD99, EMA, p16, circulating tumor DNA (ctDNA), NGS mutation analysis, tumor mutational burden, PD-L1, microsatellite instability (MSI-H) status, CA-125
Shi et al. (2017) [62]	67	F	Pancreatic cancer	Pancreas	Chemo-therapy (gemcitabine, paclitaxel albumin)	Liver, right pleura metastasis	Chemo-therapy (gemcitabine, paclitaxel albumin), tyrosine kinase inhibitor, immuno-therapy	45 Gy, 15 fr	Pancreas	Metasases	1 month	GM-CSF	CA19-9
Xie et al. (2017) [63]	54	M	Renal cell carcinoma (RCC)	Left kidney	Nephrectomy	Multiple mediastinal, retro-peritoneal, bilateral cervical, pelvis LN	Tyrosine kinase inhibitors (sunitinib)	32 Gy, 4 fr	Left mediastinal LN	All metastasis	2 months	Pembrolizumab (PD-1)	Circulating tumor DNA (ctDNA)
Britschgi et al. (2018) [64]	47	M	Non-small-cell lung carcinoma (NSCLC)	Lung	Chemo-therapy cetuximab, RT, resection	Rretro-peritoneal + abdominal lymph node	Chemo-therapy (pemetrexed), immuno-therapy	18 Gy, 3 fr	2 retro-peritoneal LNs	Unirradiated LNs	10 weeks	Nivolumab (PD-1)	History of tobacco use
Gutkin et al. (2018) [65]	57	M	Melanoma	Left posterior arm	Excision (priamry, LN)	Liver. left upper arm	Immuno-therapy	50 Gy, 20 fr	Left posterior arm			IFN therapy, ipilimumab	BRAF mutation, V600
								54 Gy, 3 fr	2 liver metastases	Complete response	1 year		
Matsushita et al. (2018) [66]	62	M	Renal cell carcinoma (RCC)	Right kidney	Nephrectomy	Right adrenal gland, lumbar vertebrae (L4)	Tyrosine kinase inhibitors (sunitinib, axitinib), immuno-therapy	36 Gy, 12 fr	Lumbar vertebrae (L4)	Right adrenal	1.5 months	Nivolumab (PD-1)	
	71	M	Renal cell carcinoma (RCC)	Right kidney	Nephrectomy	Left parotid gland, soft tissues, lung, pancreas, right iliopsoas muscle	Interferon-α therapy, tyrosine kinase inhibitors (sunitinib, axitinib), immuno-therapy	66 Gy, 33 fr	Right iliopsoas muscle	Lung, pancreas	1.5 months	Nivolumab (PD-1)	Tumor burden
Rodriguez-Ruiz et al. (2018) [67]	68	M	Castration-resistant prostate carcinoma	Prostate	Surgery, chemotherapy	Mediastinal + inguinal LN, liver, bone, lung	Chemo-therapy, immuno-therapy	24 Gy, 3 fr	Prostate, inguinal LNs	Mediastinal + retroperitoneal lesions	6 months	Poly ICLC, DC vaccine	PSA serum concentration
Tsui et al. (2018) [68]	65	F	Melanoma	Maxillary gingiva + hard palate	Resection	Floor of mouth, right neck, lung, cervical LN	Immuno-therapy	50 Gy, 20 fr	Primary tumor			Pembrolizumab (PD-1)	
								24 Gy, 3 fr	Neck	Lung, mouth	1 month		
van Gysen et al. (2018) [69]	66	F	Renal cell carcinoma (RCC)	Kidney (right)	Nephrectomy, TKI, immuno-therapy	Abdominal mass. retroperitoneal + peritoneal LNs, lung	Tyrosine kinase inhibitors (sunitinib, axitinib)	36 Gy, 12 fr	Abdominal mass	Lung	1 month	Nivolumab (PD-1)	
Wight et al. (2018) [70]	24	M	Hodgkin lymphoma	Left axillary LN	BEAM conditioning (carmustine, etoposide, cytarabine, melphalan)	lung, widespread lymph-adenopathy	BEAM conditioning (carmustine, etoposide, cytarabine, melphalan), immuno-therapy	20 Gy	Right axillary LN			Nivolumab (PD-1)	
								36 Gy	Infra-diaphrag-matic sites	Left pulmonary hilar, left parotid, right cervical nodal areas	1.5 month		
Xu et al. (2018) [71]	69	M	Merkel cell carcinoma	Right upper back	Surgical excision, RT	LNs, peripancreatic abdominal mass, left adrenal nodule	Immuno-therapy	50 Gy, 25 fr	Right axilla, posterior chest wall			Pembrolizumab (PD-1)	CK20
								8 Gy, 1 fr	Peripan-creatic abdominal mass, left adrenal nodule, omental nodule, enlarged para-aortic LNs	Hypermetabolic malignancy	12 months		
	72	M	Merkel cell carcinoma	Left thigh	Surgical excision	Left inguinal adenopathy, bilateral inguinal LNs, supra-clavicular, mediastinal, hilar, upper abdominal nodal	Immuno-therapy	8 Gy, 1 fr	Mediastinal + right hilar LN	Upraclavicular + abdominal LN, mediastinum, bilateral hila, left inguinal region	4 months	Pembrolizumab (PD-1)	
Zhao et al. (2018) [72]	65	M	Squamous cell carcinoma (SCC)	Esoph-agus	Esophagec-tomy	LNs (left retro-peritoneal, pelvic)	Chemo-therapy (cisplatin, docetaxel), immuno-therapy	42 Gy, 6 fr	LNs (left retro-peritoneal)	Non -irradiated LN (pelvic)	2 months	Pembrolizumab (PD-1)	ctDNA, mutant allele frequency (MAF)
Abbas et al. (2019) [73]	69	M	Urothelial carcinoma	Bladder	Chemo-therapy (gemcitabine, carboplatin)	Left internal iliac, para-aortic LN	Chemo-therapy, immuno-therapy	30 Gy, 12 fr	Bladder left iliac LN	All sites	4 months	Nivolumab (PD-1)	History of tobacco use, PD-L1
Bitran et al. (2019) [74]	62	F	Adeno-carcinoma (lung)	Lung (left)	Chemo-therapy (carboplatin, pemetrexed)	Left adrenal	Chemo-therapy (carboplatin, pemetrexed), immuno-therapy	27 Gy, 9 fr	Left lung	Left adrenal	7 months	Nivolumab (PD-1)	Epidermal growth factor receptor (EGFR), anaplastic lymphoma kinase (ALK), hemoglobin
Choi et al. (2019) [75]	67	M	Squamous cell carcinoma (SCC)	Lower lip	Resection	Hilar nodes, liver, peritoneum, right parotid, right neck, mediastinum, left hilum, posterior neck subcutaneous tissue, lung, left adrenal	Immuno-therapy, cobimetinib	Post-operative	Resection margin			Atezolizumab (PDL1)	
								45 Gy, 5 fr	Right sub-mandibular + neck nodes	Abdomen, chest	13 months		
D’Andrea et al. (2019) [76]	42	F	Melanoma	Right upper skin of back	Surgical excision	Upper-right retro pectoral region of chest wall, right ovary, brain axilla	Immuno-therapy	30 Gy, 15 fr	Whole brain	Chest, axilla	3 weeks	MAPK kinase (MEK) inhibitor	BRAF mutation, RBI mutation, ERCC1, MLH1, MSH2, MSH6, PMS2, TUBB3, PDL-1, TrK A/B/C, MGMT expression
Garelli et al. (2019) [77]	54	M	Pulmonary large cell neuro-endocrine carcinoma	Lung (right upper lobe)	Four cycles of chemotherapy (pemetrexed, cisplatin, bevacizumab)	Bilateral adrenal metastases	Chemo-therapy, immuno-therapy	30 Gy, 10 fr	Second + third thoracic vertebrae	Partial regression of lung tumor + adrenal metastases	4 months	Nivolumab (PD-1)	PD-L1, EGFR, or ALK mutations
	64	M	Adeno-carcinoma (lung)	Lung (left upper lobe)	Nabpaclitaxel/carboplatin with atezolizumab	Contralateral LN, brain, ocular	Chemo-therapy, immuno-therapy	30 Gy, 10 fr	Brain	Complete remission of lung + mediastinal tumor masses	4 months	Atezolizumab (PDL1)	
	70	M	Adeno-carcinoma (lung)	Lung (middle lob)	n/a	LN, brain	Immuno-therapy	30 Gy, 10 fr	Brain	Partial regression of lung tumor	2 weeks	Pembrolizumab (PD-1)	
Gounder et al. (2019) [78]	25	F	Chordoma	Sacrum	n/a	Lung	EZH2 inhibitor (tazemetostat)	70 Gy, 35 fr	Sacrum	Lung	4 months	Nivolumab (PD-1)	Next-generation sequencing, tumor mutation burden
Grimaux et al. (2019) [79]	78	M	Renal cell carcinoma (RCC)	Kidney	Immuno-therapy	Pulmonary + costal metastasis, cutaneous, oral + genital blisters,	Immuno-therapy	30 Gy, 10 fr	Costal metastases	Lung	1 month	Nivolumab (PD-1)	
Ishiyamal et al. (2019) [80]	68	F	Renal pelvic cancer	Left kidney	Nephroureterectomy + regional LN dissection, chemotherapy (cisplatin + gemcitabine)	Bladder, paraaortic LN, left subclavian LN, right renal hilum LN, left back (local recurrence in surrounding muscles)	Chemo-therapy	30 Gy, 10 fr	Left back	Reductiomn 2 paraaortic LNs, right renal hilar LN	2 months	Pembrolizumab (PD-1)	
							Immuno-therapy			2 non-irradiated lesions stable (paraaortic LN, right renal hilum LN)	21 months		
Lin et al. (2019) [81]	71	M	Adeno-carcinoma (lung)	Lung (right lobe)	Chemo-therapy	Brain, right lower lung, left lower lobe	Chemo-therapy, immuno-therapy	48 Gy, 8 fr	Brain	Lung, LNs (mediastinum)	4 months	Atezolizumab (PDL1)	
Liu et al. (2019) [82]	52	F	Intra-hepatic cholangio-carcinoma	Liver	RT	LN in hepatic hilar + retro-peritoneum	Aapatinib + lenvatinib	55Gy, 5 fr	Right hepatic lobe	LN in hepatic hilar + retroperitoneum	1 month	Nivolumab (PD-1)	Whole-exome sequencing, tumor mutation burden, PD-L1, ERBB2, HBV infection
	59	M	Intra-hepatic cholangio-carcinoma	Liver	Resection of middle hepatic lobe	Left + right lobes, hepatic hilar + retroperitoneal LN	Lapatinib	52 Gy, 4 fr	Right hepatic lobe	Hepatic hilar + retroperitoneal LN	2 to 5 months	Pembrolizumab (PD-1)	
	51	M	Intra-hepatic cholangio-carcinoma	Liver	Resection in left hepatic lobe	Right lobe hepatic hilar + retro-peritoneal lymph node	Chemo-therapy, endostatin	52 Gy, 4 fr	Left hepatic lobe, left retroperitoneal LN	Intrahepatic LN	1 month	Pembrolizumab (PD-1)	
Moran et al. (2019) [83]	71	M	Melanoma	Unknown	n/a	Lung, pelvis, omental mass, bilateral hilar nodes	Immuno-therapy	50 Gy, 5 fr	left lung	Pelvis	1 month	Ipilimumab (CTLA-4), Nivolumab (PD-1)	S100, Melan A, TTF1, P63, CK7/20, BRAF mutations
Qin et al. (2019) [84]	21	M	Nodular sclerosing Hodgkin’s lymphoma	n/a	Different chemotherapy regimes	Multiple sites of adenopathy, osseous lesions	Chemo-therapy, stell cell trans-plantation, immuno-therapy	20 Gy, 5 fr	L2, left iliac crest	Complete response	4 months	Nivolumab (PD-1)	PD-L1 expression, gene mutations, tumor mutation burden, hemoglobin, alkaline phosphatase, telomere FISH
	34	M	Nodular sclerosing Hodgkin’s lymphoma	n/a	Chemo-therapy (ICE)	Preaortic + pelvic LNs	Chemo-therapy (ICE), stell cell trans-plantation, immuno-therapy	36 Gy, 20 fr	Pelvic LNs	Complete response	1 year		
	23	M	Nodular sclerosing Hodgkin’s lymphoma	n/a	ABVD chemotherapy	Mediastinum, multiple LNs	ABVD chemo-therapy, immuno-therapy	40 Gy, 20 fr	Cervical + mediastinal LNs	Complete response	5 months	Nivolumab (PD-1)	
Shinde et al. (2019) [85]	75	M	Head + neck squamous cell carcinoma	Left neck, hypo-pharynx + oropharynx	Immuno-therapy	Left lung	Immunotherapy	14.8 Gy, 4 fr	Neck	Lung	2 weeks	Ipilimumab (CTLA-4), Nivolumab (PD-1)	History of tobacco use
								13.2 Gy, 4 fr	Margin				
Suzuki et al. (2019) [86]	30	F	Renal cell carcinoma (RCC)	Left kidney	Nephrectomy	Lung, right ovarian, pelvic + lumber vertebrae, hilar LN, mediastinal LNs, liver, left iliac bone	Sunitinib, axitinib	60 Gy, 30 fr	Mediastinal LNs			Nivolumab (PD-1)	LDH levels
								35 Gy, 5 fr (brachy-therapy)	Left iliac lesion left internal iliac LN	Lumbar vertebrae (L4)	3 months		
Trommer et al. (2019) [87]	n/a	n/a	Melanoma	Left thigh	Resection	Lung, paraaortal LN, brain	Immuno-therapy	20 Gy, 1 fr	Brain			Pembrolizumab (PD-1)	
								50 Gy, 2 fr	Brain	Lung	1 month		
	n/a	n/a	Melanoma	Left knee	Resection	Perirenal region, brain	Immuno-therapy	40 Gy, 2 fr	Whole brain			Pembrolizumab (PD-1)	
								54 Gy, 3 fr	Popliteal fossa + lower left leg, brain	Perirenal region	1.5 month		
								20 Gy, 1 fr	Brain				
	n/a	n/a	Melanoma	n/a	n/a	Lung, brain	Immuno-therapy	20 Gy, 1 fr	Brain			Pembrolizumab (PD-1)	
								20 Gy, 1 fr	Brain				
								20 Gy, 1 fr	Brain	Lung	5 weeks		
	n/a	n/a	Non-small cell lung carcinoma (NSCLC)	Lung	n/a	Suprarenal glands, brain, right femur, os. sacrum left os. ischiadicum	Immuno-therapy	27 Gy, 3 fr; 20 Gy	Brain			Nivolumab (PD-1)	
								30 Gy, 3 fr	Right femur	Suprarenal glands	1 month		
								30 Gy, 3 fr	Os. sacrum, left os. ischiadicum				
	n/a	n/a	Non-small cell lung carcinoma (NSCLC)	Lung	n/a	Cervival + left super-vlavicular, mediastinal Ln, left hilar, axillar LN, intracarinal LN	Immuno-therapy	54 Gy, 2 fr	Cervival + left supervlavicular	axillar LN	6 months	Nivolumab (PD-1)	
	n/a	n/a	Non-small cell lung carcinoma (NSCLC)	Lung	n/a	Supra- + infra-clavicular lymph drainage area, 3rd right rib, right iliac sacral joint, left inguinal, lung	Immuno-therapy	50.4 Gy, 1.8 fr	Supra- + infra-clavicular lymph drainage area			Nivolumab (PD-1)	
								30 Gy, 3 fr	3rd right rib, right iliac sacral joint, left inguinal				
								20 Gy, 1 fr	Left occipital	Lung	7 weeks		
	n/a	n/a	Renal cell carcinoma (RCC)	Kidney	n/a	Left os. ilium, left + right hip, right os. pubis, thoracic vertebra, lumbar vertebra, mediastinal LN, hilar, pleural	Immuno-therapy	36 Gy, 3 fr	Left os. ilium	mediastinal LN	2 months	Nivolumab (PD-1)	
								30 Gy, 3 fr	Left + right hip, right os. pubis				
								30 Gy, 3 fr	Thoracic vertebra, lumbar vertebra				
Yaguchi et al. (2019) [88]	63	M	Pulmonary pleo-morphic carcinoma	Lung (right upper lobe)	Chemo-therapy (carboplatin, paclitaxel)	Brain, bone + pleural metastases	Chemo-therapy (carboplatin, paclitaxel), immuno-therapy	30 Gy, NR	Right hip joint over right femur	Pleura, brain + bone		Nivolumab (PD-1)	History of tobacco use, PD-L1 expression of tumor, EGFR mutation, ALK
Forner et al. (2020) [89]	57	M	Head + neck squamous cell carcinoma	Left frontal sinus + ethmoid sinuses	Resection, chemotherapy	Right lower lobe, left cervical LNs, subcarinal LN, extraconal intraorbital lesion	Chemo-therapy (cisplatin)	66 Gy, 33 fr				Nivolumab (PD-1)	Medical history
								30 Gy, 5 fr	Intraorbital mass	Thorax metastases, lung	1 month		
Hori et al. (2020) [90]	40	F	Renal cell carcinoma (RCC)	Left kidney	Nephrectomy	Lung, right supra-clavicular + para-aortic LN	Interferon-α, axitinib, everolimus, pazopanib	30 or 40 Gy, 10 fr	Right supra-clavicular + para-aortic LN	Lung	6 months	Nivolumab (PD-1)	HLA class1, CD8, PD-L1 expression of tumor
Igarashi et al. (2020) [91]	74	M	Melanoma	Maxilla	Maxillary resection	Cervical LNs, brain, spleen, liver	Immuno-therapy	30 Gy, 10 fr	Whole brain	Liver, spleen	2 months	Nivolumab (PD-1)	BRAF mutation
Kuhara et al. (2020) [92]	69	F	Un-resectable gastric cancer (UGC)	Lower portion of stomach	Chemo-therapy (S-1 plus oxaliplatin (SOX) + Herceptin (HER))	Para-aortic, mediastinal, right iliac + Virchow LNs, bilateral subclavian + mediastinal LNs, left axillary LNH, peritoneal metastasis, left iliac, right diaphragm LN, left adrenal gland	Chemot-herapy (S-1 plus oxaliplatin (SOX) + Herceptin (HER)), immuno-therapy	55 Gy, 22 fr	Neck + mediastinum	Bilateral subclavian + mediastinal LNs	13 months	Nivolumab (PD-1)	EGF-2
								50 Gy, 10 fr	Left adrenal	Para-aortic, bilateral iliac, right diaphragm LNs			
Levitin et al. (2020) [93]	83	M	Renal cell carcinoma (RCC)	Left kidney	Pazopanib, SBRT 40Gy, 5 fr	Right lung left femur, brain, mediastinal lymphadenopathy, pelvis	Receptor tyrosine kinase inhibitor (pazopanib), immuno-therapy	GKS 20 Gy	Brain	Regression metastases	2 years	Nivolumab (PD-1)	
Nakajima et al. (2020) [94]	56	F	Clear cell carcinoma	Kidney	Nephrectomy	Left lung hilum, subcutaneous + lung metastases, right renal, right iliac bone	Receptor tyrosine kinase inhibitor (pazopanib, axitinib), immuno-therapy	30 Gy, 10 fr	Right iliac bone	Lung, right kidney, subcutaneous tissue	5 months	Nivolumab (PD-1)	
										Primary tumor	9 months		
Sohal et al. (2020) [95]	66	M	Melanoma	Right nasal cavity	RT	Left proximal humerus, upper thoracic spine, liver, lateral left fifth rib, third, eighth, eleventh thoracic vertebral bodies	Immuno-therapy	25 Gy, 5 fr	Nose	All metastasis	4 months	Nivolumab (PD-1)	Cancer history, transplantation history, BRAF, NRAS mutation
Wang et al. (2020) [96]	57	M	Squamous cell carcinoma (SCC)	Right upper lung	Resection, adjuvant RT, chemotherapy	Pancreas, metastatic nodules in left lower lobe	Chemo-therapy (gemcitabine, cisplatin)	55 Gy, 25 fr	Resection margin			Pembrolizumab (PD-1)	PD-L1, ALK genetic status, history of tobacco use, whole-exome sequencing
								60.2 Gy, 28 fr	Pancreas	Left lung nodules	1 month		
Watanabe et al. (2020) [97]	69	F	Melanoma (BRAF-wild-type)	Upper left leg	Resection, also sentinel LNs	Left groin LN, liver, muscle in upper left leg, left groin	Immuno-therapy, corticosteroids, adjuvant IFN-α therapy	45 Gy, 3 fr	Liver lesions	Left groin LN, liver, muscle in upper left leg, left groin	8 weeks	Nivolumab (PD-1)	CD8 cells, PD-1, PD-L1, TIM-3, LAG-3, TOX, IFN-γ, perforin, granzymes, IL-2, TNF-α, DCXCL9, CXCL10, CCL, CXCL13, IDO1, CD4+ Tregs, arginase, MHC I, MHC II, APCs, β2-microglobulin, Ki67, CD3+ cells, tumor burden, FoxP3, exhausted T cells, BRAF-mutation
	77	M	Melanoma (BRAF-wild-type)	Right breast region	Resection, also sentinel lymphnodes	Liver, lung, abdominal LN	Immunotherapy	45 Gy, 3 fr	Liver metastases	Stable	6 weeks	Pembrolizumab (PD-1)	
								60 Gy, 8 fr	LN, liver	Other liver lesions, lung	1 month		
Hotta et al. (2021) [98]	42	F	Adeno-carcinoma (lung)	Lung (left upper lobe)	EGFR tyrosine kinase inhibitor, steroid	Bone, brain, thoracic spine	EGFR tyrosine kinase inhibitor, steroid	30 Gy, 10 fr	Whole brain	Lung	4 days	Atezolizumab (PDL1)	EGFR. PD-L1, CEA
								36 Gy, 12 fr	Thoracic spine	Lung	2 weeks		
Mampuya et al. (2021) [99]	72	M	Malignant pleural mesothe-lioma	Right lung	Chemo-therapy (carboplatin, pemetrexed),VEGF-A inhibitor (bevacizumab)	Para-mediastinal + retro-pharyngeal lesions	Chemo-therapy (carboplatin, pemetrexed),VEGF-A inhibitor (bevacizumab), immuno-therapy	30 Gy, 10 fr	Para-mediastinal lesions	Right lung	3 months	Pembrolizumab (PD-1)	Medical history, PD-L1, tumorinfiltrating CD3 T cells, CD8 T cells
Wong et al. (2021) [100]	n/a	n/a	Renal cell carcinoma (RCC)	Kidney	n/a	Lung	Immuno-therapy	55 Gy, 20 fr	Lung	Lung	n/a	Ipilimumab (CTLA-4), Nivolumab (PD-1)	
	n/a	n/a	Renal cell carcinoma (RCC)	Kidney	n/a	Femur, rib		25 Gy, 5 fr	Femur, rib	Nephrectomy bed	n/a	Ipilimumab (CTLA-4), Nivolumab (PD-1)	
	n/a	n/a	Renal cell carcinoma (RCC)	Kidney	n/a	Lung, adrenal		36 Gy, 3 fr	Lung	Lung, several lesions	n/a	Ipilimumab (CTLA-4), Nivolumab (PD-1)	
								30 Gy, 5 fr	Adrenal	Lung			
	n/a	n/a	Renal cell carcinoma (RCC)	Kidney	n/a	Brain, intramuscular		GKS 25Gy	Brain	Intramuscular	n/a	Ipilimumab (CTLA-4), Nivolumab (PD-1)	
	55	F	Renal cell carcinoma (RCC)	Kidney	n/a	Femur, pubic bone, liver		8 Gy, 1 fr	Femur	Liver, several lesions	n/a	Pembrolizumab (PD-1)	
								27 Gy, 3 fr	Pubic bone				
	n/a	n/a	Renal cell carcinoma (RCC)	Kidney	n/a	Lung		54 Gy, 3 fr	Lung	Lung, several lesions	n/a	Nivolumab (PD-1)	
								42 Gy, 5 fr	Lung				
								25 Gy, 5 fr	Lung				
	n/a	n/a	Renal cell carcinoma (RCC)	Kidney	n/a	Lung		50 Gy, 20 fr	Lung	Lung	n/a	Nivolumab (PD-1)	
								48 Gy, 3 fr	Lung				
								48 Gy, 3 fr	Lung				
	n/a	n/a	Renal cell carcinoma (RCC)	Kidney	n/a	Femur, lliac bone, brain, lung		20 Gy, 5 fr	Femur	Lung, several lesions	n/a	Nivolumab (PD-1)	
								24 Gy, 3 fr	Iliac bone				
								GKS 25 Gy	Brain				
	n/a	n/a	Renal cell carcinoma (RCC)	Kidney	n/a	Iliac bone, spine, lung		27 Gy, 3 fr	Iliac bone	Lung, several lesions	n/a	Nivolumab (PD-1)	
								24 Gy, 3 fr	Spine	Kidney			

Abbreviations: M: male; F: female; n/a: not available; RT: radiotherapy; LN: lymph nodes; fr: fractions; CTLA-4: cytotoxic T-lymphocyte-associated protein 4; ALC: Absolute lymphocyte counts; AEC: absolute eosinophil counts; TIA-1: TIA1 Cytotoxic Granule Associated RNA Binding Protein; Tregs: regulatory T cells; CK: cytokeratin; TTF-1: thyroid transcription factor 1; CDX2: Caudal Type Homeobox 2; CEA: carcinoembryonic antigen; MEGA3: Melanoma antigen A3; PASD1: PAS domain containing 1; DC-CIK: cytokine-induced killer cells; PD-1: programmed cell death protein 1; DC: dendritic cell; CA 19-9: carbohydrate antigen 19-9; ctDNA: circulating tumor DNA, NGS: next generation sequencing; PD-L1: PD-1 receptor-ligand 1; MSI-H: high levels of microsatellite instability; CA-125: cancer antigen 125; GM-CSF: granulocyte-macrophage colony-stimulating factor; MAF: mutant allele frequency; EGFR: epidermal growth factor receptor; ALK: anaplastic lymphoma kinase; FISH: fluorescent in situ hybridization; LDH: lactate dehydrogenase; EGF: epidermal growth factor; TIM-3: T cell immunoglobulin and mucin-domain containing-3; LAG-3: lymphocyte-activation gene 3; TOX: thymocyte selection-associated high mobility group box protein; IFN-γ: interferon gamma; TNF-α: tumor necrosis factor alpha; MHC: major histocompatibility complex; APC: antigen-presenting cells.

**Table 3:** Abscopal effect case reports in different tumor entities.

Tumor Entities	Incidence	Mortality	Cases of AbE		
	New Cases Worldwide in 2020 (% of All Sites) [147]	New Deaths Worldwide in 2020 (% of All Sites) [147]	Total	Without ICB	Under ICB
Breast	11.7%	6.9%	2	2	-
Lung	11.4%	18.0%	18	6	12
Colorectum	10.0%	9.4%	2	1	1
Prostate	7.3%	3.8%	1	-	1
Stomach	5.6%	7.7%	1	1	-
Esophagus	3.1%	5.5%	2	2	-
Thyroid	3.0%	0.4%	1	1	-
Bladder	3.0%	2.1%	2	1	1
Kidney	2.2%	1.8%	23	5	20
Melanoma	1.7%	0.6%	29	2	27

**Table 4:** Potential predictive biomarkers for abscopal effects.

Potential Predictor	Preclinical Evidence		Clinical Evidence	
Tumor protein p53	Camphausen et al. [183]	Requirement of intact p53 to induce AbE		
	Strigari et al. [184]	Non-irradiated tumors with functional p53 respond systematically to irradiation		
Calreticulin	Gameiro et al. [115]	Calreticulin expression as evidence for enhanced CTL lysis of non-irradiated tumor cells		
Hsp70	Bayer et al. [185]	Plasma levels of Hsp70 associated with tumor burden, level correlates with tumor progression and radiation-induced tumor regression		
γ-H2AX			Siva et al. [186]	γ-H2AX foci in non-irradiated PBL and hair follicles indicates AbE in normal tissue
PD-L1			Yaguchi et al. [88]	Correlation of PD-L1 expression to abscopal response
			Watanabe et al. [97]	
Tumor markers				
• SCC antigen			Joe et al. [31]	SCC Ag levels decreases due to systemic response
• S100 (melanoma)			Watanabe et al. [97]	Correlation of decrease in S100 with AbE
• CEA			Golden et al. [52]	Drastic drop to normal levels during systemic response
			Kuroda et al. [43]	Drop in plasma levels during occurrence of AbE
• CA15-3			Leung et al. [39]	Significant decrease in CA15-3 levels during abscopal reaction
• CA19-9			Bonilla et al. [41]	Reduced plasma levels during AbE
• CA125			Tomita et al. [48]	Reduced plasma levels during AbE
ctDNA abundance and tumor mutational burden (TMB)			Xie et al. [63]	Correlation of ctDNA abundance with abscopal extinction of tumor cells
			Zhao et al. [72]	ctDNA analysis for mutation monitoring associated with AbE
			Sharabi et al. [61]	Correlation of elevated TMB with AbE
Tumor antigens				
• melanoma antigen A3 (MAGEA3)			Stamell et al. [53]	Association of AB to tumor antigens with AbE
• PAS domain-containing 1 (PASD1)				
• NY-ESO-1			Postow et al. [49]	AB titers against NY-ESO-1 correlated positively with disease progression
Absolute lymphocyte count (ALC)			Antoniades et al. [9]	Correlation of irradiation-induced decrease in ALC with increase in neutrophils
			Desar et al. [28]	Monitoring changes of lymohocytes during AbE
			Kuroda et al. [43]	
			Postow et al. [49]	
			Blattner et al. [187]	
			Golden et al. [52]	Disease progression tracked by ALCs, white blood cells (WBCs) and absolute eosinophil counts (AECs)
			Grimaldi et al. [54]	ALC was increased before RT if AbE
			Chen et al. [188]	Correlation between increased ALC value after RT and AbE
T cell values			Kuroda et al. [43]	low CD8+ lymphocyte counts at the time of AbE
			Postow et al. [49]	IFNγ-producing CD8+ and CD4+ T cells decrease due to RT and increase slightly during AbE
Neutrophil-to-lymphocyte ratio			Golden et al. [189]	significantly lower baseline in non-responders
Tumor-infiltrating lymphocytes			Joe et al. [31]	Infiltration pattern of tumor reacting with AbE
			Hori et al. [121]	Infiltration of non-irradiated lesion by CD8+ T cells
			Teulings et al. [25]	Infiltration of abscopal reacting tumors with CD8+ T cells
			Mampuya et al. [99]	Moderate infiltration of CD3+ cells after occurrence of AbE
Cytokine profiles			Ohba et al. [15]	Monitoring serum concentrations of various cytokines in AbE, TNF-α serum levels increased in AbE
Exosomes			Chen et al. [188]	Circulating TEXs analysis could distinguish responders from non-responders

**Table 5:** Currently ongoing clinical trials.

Study Identifier	Study Title	Status	Condition or Disease	Therapy	RT Scheme	Cohort N	Planned Primary Outcome Measures	Planned Secondary Outcome Measures
NCT03480334	Abscopal Effect of Radiotherapy and Nivolumab in Relapsed Hodgkin Lymphoma After Anti-PD-1 Therapy (AERN)	Recruiting	Classical Hodgkin lymphoma	Nivolumab + RT	20 Gy	29	Abscopal response rate	-
NCT03396471	Study of Pembrolizumab and Concurrent Radiation in Patients With Previously Treated Carcinoma of Unknown Primary	Recruiting	Carcinoma, unspecified site	Pembrolizumab + EBRT	20-30 Gy, 5 fr	34	Abscopal response rate	Response rate
								Assess adverse events
								Progression free survival
								Overall survival
								Time-to-progression
								Disease control rate
NCT04238169	Clinical Trial Assessing the Efficacy of Abscopal Effect Induced by SBRT and Immunotherapy in Advanced NSCLC	Recruiting	Non-Small-Cell Lung Cancer (NSCLC)	(Bevacizumab +) Toripalimab + SBRT	30-50 Gy, 5 fr	60	Objective response rate	Progression free survival
								Duration of response
							Objective response of non-target lesion	Overall survival
								Incidence of adverse events
								Quality of life
NCT04873440	An Open-label, Phase I/II Study of Manganese Plus Radiotherapy in Patients With Metastatic Solid Tumors or Lymphoma	Recruiting	Solid tumor	Manganese Chloride + RT (Chemo-immuno-therapy)	standard-of-care RT or SBRT to one metastatic site	10	Proportion of subjects with an abscopal response	Disease control rate
								Progression free survival
			Lymphoma				Number of treatment-related adverse events	Overall survival
								Number of participants with laboratory test abnormalities
NCT04168320	SBRT-based PArtial Tumor Irradiation of HYpoxic Segment (SBRT-PATHY)	Recruiting	Unresectable malignant solid neoplasm bulky tumors	SBRT	SBRT-based PArtial Tumor irradiation targeting HYpoxic segment	30	Bystander and abscopal effects	Overall survival
								Progression free survival
								Patient-reported outcome
								Incidence of adverse events
								Response evaluation criteria in solid tumors
								Timing
NCT03449238	Pembrolizumab And Stereotactic Radiosurgery (Srs) Of Selected Brain Metastases In Breast Cancer Patients	Recruiting	Metastatic breast cancer	Pembrolizumab + Stereotactic Radiosurgery	n/a	41	Tumor response for non-irradiated brain lesions	Number of participants with abscopal response
			Brain metastases				Correlation of abscopal responses with the radiation dose received	
							Overall survival	
NCT04245514	Multimodality Treatment in Stage III Non-small Cell Lung Cancer (NSCLC)	Recruiting	Non-Small-Cell Lung Cancer (NSCLC)	Durvalumab + RT	40 Gy, 20 fr	90	Event-free survival	Recurrence-free survival after resection
								Overall survival
					25 Gy, 5 fr			Objective response
								Pathological complete response
					24 Gy, 3 fr			Major pathological response
								Complete resection
NCT04530708	Addition of Radiotherapy to Standard Medical Treatment for Stage IV NSCLC (MARS)	Recruiting	Non-Small-Cell Lung Cancer (NSCLC)	Thoracic RT vs. standard of care	36 Gy	162	Difference in quality of life	Overall survival
								Progression free survival
								Toxicity of esophagitis, pneumonitis, dyspnea, fatigue, cough
NCT04212026	Irreversible Electroporation (IRE) Followed by Nivolumab in Patients With Metastatic Pancreatic Cancer.	Recruiting	Metastatic pancreatic cancer	Nivolumab	n/a	15	Overall response rate (ORR) of the reference liver metastasis	ORR of primary tumor site (pancreas)
								ORR of IRE-treated liver metastasis
								Progression free survival (PFS)
								Overall survival
								Adverse events
NCT03474497	IL-2, Radiotherapy, and Pembrolizumab in Patients Refractory to Checkpoint Blockade	Recruiting	NSCLC, Head and neck squamous cell carcinoma,	IL-2 + Pembrolizumab + RT	24 Gy, 3 fr, palliative regimen	45	Abscopal response rate	Maximum tolerated dose
			Metastatic melanoma					
			Metastatic RCC					
NCT03548428	Stereotaxic Body Irradiation of Oligometastase in Sarcoma (Stereosarc)	Recruiting	Sarcoma	Atezolizumab + SBRT	3 to 5 fr depending on tumor size	103	Progression-free survival (PFS) rate	PFS by immune response criteria
								Ratio PFS after RT/PFS during previous treatment
								Objective response rate
								Toxicity of treatment
								Overall survival
								Quality of life
								Evaluation of the cost of treatment
								Rate of PET-CT at inclusion
								Impact of biomarkers on PFS or response rate
								Developing mathematical models
NCT03927898	Phase II Study of Toripalimab Plus Stereotactic Body Radiotherapy in Colorectal Cancer Patients With Oligometastasis	Recruiting	Metastatic colorectal cancer	Toripalimab + SBRT	SBRT (BED >80Gy) to oligometastatic lesions	40	1 year progression free survival (PFS)	Acute adverse events
								Objective response rate
								2 year local control rate
								2 year overall survival
								T cell receptor repertoire/T cell clones in blood
								Expression of PD-1, Ki-67 on T cell
								Expression of PD-L1 on exosomes in blood
								Expression of PD-L1 on circulation tumor cell
NCT03774732	PD-1 Inhibitors and Chemotherapy With Concurrent Irradiation at Varied Tumour Sites in Advanced Non-small Cell Lung Cancer (NIRVANA-LUNG)	Recruiting	Non-Small-Cell Lung Cancer (NSCLC)	Pembrolizumab + Chemotherapy + Radiotherapy	at least 18 Gy in 3 fr	460	Overall survival	Tumour response
								Progression free survival
								Local and distant controls in irradiated patients
				Pembrolizumab + Chemotherapy				Quality of life
								Acute/late toxicities
NCT04299646	Study Assessing Stereotactic Radiotherapy in Therapeutic Strategy of Oligoprogressive Renal Cell Carcinoma Metastases (GETUG-StORM-01)	Recruiting	Metastatic RCC	Stereotactic RT + systemic treatment	n/a	114	Progression free survival	Treatment-related adverse events
								Local control rate
								Overall control rate
NCT03316872	Study of Pembrolizumab and Radiotherapy in Liver Cancer	Recruiting	Hepatocellular carcinoma	Pembrolizumab + SBRT	n/a	30	Overall response rate	Response rate in non-irradiated tumor lesions
								Progression free survival rate
								Overall survival rate
NCT03277482	Durvalumab, Tremelimumab + Radiotherapy in Gynecologic Cancer	Recruiting	Recurrent gyneco-logical cancer	Durvalumab + Tremelimumab + RT	n/a	32	Maximum tolerated dose	Overall response rate
			Metastatic cervical cancer					Local response rate
			Metastatic ovarian cancer					Abscopal response rate
			Metastatic vaginal cancer					Response duration
			Metastatic vulvar cancer					Progression free survival
			Metastatic endometrial cancer					
NCT03085719	Targeting PD-1 Therapy Resistance With Focused High or High and Low Dose Radiation in SCCHN	Recruiting	Head and neck cancer	High dose radiation + Pembrolizumab	n/a	26	Overall response rate	Overall survival
								Progression free survival
								Treatment-related adverse events
								Immune-related response
								Local response
								Clinical benefit rate
								Abscopal response
NCT03176173	Radical-Dose Image Guided Radiation Therapy in Treating Patients With Metastatic Non-small Cell Lung Cancer Undergoing Immunotherapy	Recruiting	Non-Small-Cell Lung Cancer (NSCLC)	Immunotherapy + Image Guided RT	n/a	85	Progression free survival	Change in ctDNA levels
								Immune marker levels from peripheral blood
								Acute and late toxicity
								Overall survival
								Patterns of response and progression
								Time to discontinuation of study
NCT04221893	Radiation Therapy for the Treatment of Metastatic Gastrointestinal Cancers	Recruiting	Esophageal adenocarcinoma	RT	n/a	28	Overall response rate	Progression free survival
			Esophageal squamous cell carcinoma					Overall survival Determine local control in radiated lesion(s)
			Gastric cancer					Tumor measurement change
			Adenocarcinoma of gastroesophageal junction					New metastatic lesions Adverse events
NCT03385226	A Trial Assessing the Effect of Pembrolizumab Combined With Radiotherapy in Patients With Relapsed, Refractory, Specified Stages of Cutaneous T-cell Lymphoma (CTCL) Mycosis Fungoides (MF)/Sezary Syndrome (SS) (PORT)	Recruiting	Cutaneous T cell lymphoma	Pembrolizumab + RT	12 Gy, 3 fr	46	Overall response (global assessment)	Response and duration
								Safety and toxicity
								Progression free survival
								Overall survival
								Abscopal effect rate
								Changes in immune status
								Plasma HMGB-1 levels
								Functional analysis of isolated cell populations
								Assessment of diversity and clonality of T cells
								Immune signatures for responders and non-responders
								Tumor-infiltrating lymphocyte-specific neo-antigens
								Expression of immunological checkpoints
								Investigation of tumor immune microenvironment
NCT02858869	Pembrolizumab and Stereotactic Radiosurgery for Melanoma or Non-Small Cell Lung Cancer Brain Metastases	Recruiting	Metastatic malignant	Pembrolizumab + Stereotactic Radiosurgery	SRS 6 Gy, 9 Gy or 18–21 Gy	30	Proportion of dose-limiting toxicities	Absolute cell counts for pre and post-treatment serum immune biomarkers
			Neoplasm in the brain					
			Metastatic melanoma					Overall response
			Mucosal melanoma					Overall survival
			Ocular melanoma					Rate of anywhere intra-cranial failure
			Non-Small Cell Lung Cancer					Rate of leptomeningeal disease
			Skin melanoma					Rate of local recurrence
			Melanoma of unknown primary					Rate of symptomatic radiation necrosis
NCT02830594	Pembrolizumab and Palliative Radiation Therapy in Treating Patients With Metastatic Esophagus, Stomach, or Gastroesophageal Junction Cancer	Active, not recruiting	Gastric and esophageal adeno-carcinoma	EBRT + Pembrolizumab	n/a	14	Tumor-infiltrating cytotoxic T cells and circulating cytotoxic T cells	Incidence of adverse events
			Gastric and esophageal squamous cell carcinoma				Immunosuppressive Tregs in non-irradiated sites	Overall response rate
			Gastroesophageal junction adeno-carcinoma				MDSCs in non-irradiated sites	Progression free survival
			Metastatic malignant neoplasm in the stomach					Overall survival
NCT03469713	Nivolumab Plus Stereotactic Body Radiotherapy in II and III Line of Patients With Metastatic Renal Cell Carcinoma (NIVES)	Active, not recruiting	Metastatic renal cancer	Nivolumab + SBRT	30 Gy in 3 consecutive fractions	69	Objective response rate (ORR)	Progression free survival
								Overall survival
								ORR of irradiated and non-irradiated metastases and duration of response
								Incidence, nature and severity of adverse event
								Analysis of expression of PD-L1
								Analysis of the genetic background of tumor
								Analysis of the immuno-modulation
								Identification of somatic mutations associated with acquired resistance to checkpoint inhibitors
NCT03453892	Investigation of the Timely-coordinated Therapy of Patients With Metastatic Cancer by Radiotherapy Together With Immune Checkpoint Inhibition (ST-ICI)	Active, not recruiting	Metastatic cancer	RT + Ipilimumab	n/a	150	Systemic and local response of detected metastases	Detection of adverse events
								Documentation of corticoid prescription
				Nivolumab + Pembrolizumab + RT			Change of circulating immune cells by deep immunophenotyping	Overall survival
								Progression free survival
NCT03322384	Phase I/II Trial of Epacadostat, Intralesional SD101, Radiotherapy in Patients With Lymphoma	Active, not recruiting	Advanced solid tumors	Epacadostat + SD-101 + RT	24 Gy, 3 fr	20	Maximum tolerated dose	Abscopal response rate
			Lymphoma		20 Gy, 5 fr		Oncidence of treatment-emergent adverse events	
NCT03539198	Study of Proton SBRT and Immunotherapy for Recurrent/Progressive Locoregional or Metastatic Head and Neck Cancer	Active, not recruiting	Head and neck cancer	Proton SBRT + Nivolumab	35–45 Gy, 5 fr	91	Objective response rate	Local control rate, overall survival
								Progression free survival
								Time to progression
								New development of distant metastasis
								Quality of Life
								Predictive and prognostic biomarker
NCT03042156	Immunotherapy And Palliative Radiotherapy Combined In Patients With Advanced Malignancy	Active, not recruiting	Advanced cancer	Palliative RT	n/a	30	Number of patients showing toxicity	In-field response on imaging and evidence of out of field (abscopal) response
								Biomarkers analyses as indicator of abscopal response
								Patient-reported outcome
								Inflammatory and radiation sensitivity signatures
NCT03483012	Atezolizumab + Stereotactic Radiation in Triple-negative Breast Cancer and Brain Metastasis	Active, not recruiting	Breast cancer	Atezolizumab + stereotactic radiosurgery (SRS)	n/a	45	Progression free survival	Extracranial objective response rate
								Abscopal response rate
								Clinical benefit rate
								Overall survival
								Patient-reported outcome
								Development of radiation necrosis
								Assessed neurological evaluation
								Dose-limiting toxicity
NCT02888743	Durvalumab and Tremelimumab With or Without High or Low-Dose Radiation Therapy in Treating Patients With Metastatic Colorectal or Non-small Cell Lung Cancer	Active, not recruiting	Metastatic colorectal	Tremelimumab + Durvalumab	n/a	180	Overall response rate	Progression free survival
								Overall survival
								Objective response
			Carcinoma metastatic lung					Incidence of adverse events
				Tremelimumab + Durvalumab + RT				Local control rate and abscopal response rates
			Non-Small Cell Carcinoma					Prognostic effect of PD-L1 expression
								Prognostic effect of T-cell infiltration
								Symptomatic adverse events
NCT02587455	Pembrolizumab and Palliative Radiotherapy in Lung (PEAR)	Active, not recruiting	Thoracic tumours	Pembrolizumab + RT	n/a	48	Toxicity rate	Progression free survival rates
								Overall survival rates
								Duration of clinical benefit
							Maximum tolerated dose	Response rates
								Assessing individual lesion response
								Identify biomarkers and correlate with clinical benefit
NCT03601455	Radiation Therapy and Durvalumab With or Without Tremelimumab in Treating Participants With Unresectable, Locally Advanced, or Metastatic Bladder Cancer	Active, not recruiting	Bladder urothelial carcinoma	RT + Durvalumab	n/a	13	Incidence of adverse events	Local control at primary irradiate site
								Pathologic complete rate of irradiated tumor
								Overall response rate
								Abscopal response
								Duration of response
								Disease-specific survival
				RT + Durvalumab + Tremelimumab			Progression free survival	Overall survival
								Incidence of adverse events
								Immune cell subsets and PD-L1 in tumor biopsies
								Gene signature biomarker
								Circulating immune cell subsets
								Circulating and tumor-infiltrating T-cell receptor repertoire

Abbreviations: RT: radiotherapy; NSCLC: Non-Small-Cell Lung Cancer; fr: fractions; SBRT: stereotactic body radiotherapy; n/a: not available; ORR: overall response rate; IRE: irreversible electroporation; PFS: progression free survival; RCC: renal cell carcinoma; PET-CT: positron emission tomography; CT: computed tomography; BED: radiation biologically effective dose; PD-1: programmed cell death protein 1; PD-L1: PD-1 receptor-ligand 1; ctDNA: circulating tumor DNA; HMGB1: high-mobility group box 1; SRS: stereotactic radiosurgery.

## Data Availability

Data have been included within the article. Therefore, no additional data file is required.
